# Association of nutrition, water, sanitation and hygiene practices with children’s nutritional status, intestinal parasitic infections and diarrhoea in rural Nepal: a cross-sectional study

**DOI:** 10.1186/s12889-020-09302-3

**Published:** 2020-08-15

**Authors:** Akina Shrestha, Jeanne Six, Dikshya Dahal, Sara Marks, Regula Meierhofer

**Affiliations:** 1 Eawag, Swiss Federal Institute of Aquatic Science and Technology, Überlandstrasse 133, 8600 Dübendorf, Switzerland; 2 Kathmandu University School of Medical Sciences, Dhulikhel Hospital, GPO Box, Kathmandu, 11008 Nepal; 3 Aquatic Ecology Centre, School of Science, Kathmandu University, P.O. Box 6250, Dhulikhel, Nepal

**Keywords:** Child health, Drinking water quality, Sanitation and hygiene, Diarrhoea, Intestinal parasitic infections, Nepal, Undernutrition

## Abstract

**Background:**

Providing universal access to safe water, sanitation and hygiene (WASH) in remote Nepal remains challenging. We investigated WASH conditions and their association with children’s nutritional status, intestinal parasitic infections and diarrhoea.

**Methods:**

Data was collected through a cross-sectional survey of 1427 households, including questionnaires, observations, stool analysis, anthropometry, water quality measurements, and assessment of clinical signs of nutritional deficiencies.

**Results:**

We found 55.5% of children were undernourished, 63.9% had clinical signs of nutritional deficiencies, 51.1% had intestinal parasitic infections and 52.2% had diarrhoea. Multivariate mixed logistic regression analysis revealed a statistically significant negative association between undernutrition and socio-economic level, with adjusted odds ratios (AOR) of 0.70 (95%-CI = 0.43–1.11) and 0.43 (95%-CI = 0.25–0.75) for high and intermediate levels compared to the lowest level. Undernutrition was negatively associated with regular deworming of children (AOR = 0.44, 95% CI = 0.20–0.94), food supplements (AOR = 0.57, 95% CI = 0.38–0.84), household’s own food production (AOR = 0.67, 95% CI = 0.46–0.97) and personal hygiene (AOR = 0.83, 95% CI = 0.51–1.35). Nutritional deficiency was negatively associated with handwashing after cleaning a baby’s bottom (AOR = 0.60, 95% CI = 0.40–0.92) and cleanliness of caregiver’s hands (AOR = 0.61, 95% CI = 0.41–0.89) and positively associated with keeping animals inside the house overnight (AOR = 1.71, 95% CI = 1.17–2.51) and the presence of total coliforms in the drinking water source (AOR = 10.44, 95% CI = 1.61–67.4). Diarrhoea was positively associated with intermittent water supply (AOR = 2.72, 95% CI = 1.18–6.31) and the presence of a mud floor (AOR = 2.29, 95% CI = 1.20–4.37) and negatively associated with cleanliness of the toilet (AOR = 0.68, 95% CI = 0.47–0.98), and the cleanliness of children’s hands (AOR = 0.62, 95% CI = 0.40–0.96).

**Conclusions:**

Our study found, more than half of the survey children were in a critical health condition. Results suggest that child health improvements are dependent on multiple public health improvements, including providing better nutrition, promoting adequate hygiene behaviour, such as handwashing, keeping the latrines clean, keeping the household environment free from animal faeces and assuring a reliable supply of safe water.

## Background

Children in low-income countries face a range of interrelated problems, such as poor nutrition, inadequate water, sanitation, and hygiene (WASH), consequent infections, and growth and development impairments [1]. Globally, a total of 297,000 WASH-attributable diarrhoea deaths occur per year among children under 5 years, every fifth child’s growth is stunted, one in thirteen is wasted, and every seventh child is underweight. Nearly 90% of these cases occur in South Asia and Sub-Saharan Africa [2–4]. Furthermore, two billion people worldwide are infected with intestinal parasites, with the highest burden of this disease among children in resource-poor settings [5–7]. Studies have shown that infections with intestinal parasites among children are associated with stunting, physical weakness, and low educational performance [6, 8, 9].

Nutrition is closely interlinked with multiple determinants [10, 11]. While malnutrition is directly associated with insufficient dietary intake, underlying contributing factors, such as lack of access to safe water and sanitation, result in such recurrent infectious diseases as diarrhoea and intestinal worms. These parasites interfere with the digestive process by competing with the host for nutrients and inhibiting the absorption of nutrients, leading to compromised immunity [10, 11]. It is estimated that up to 45% of global malnutrition-related child deaths could be prevented by improving WASH conditions and practices [4, 12, 13].

Even though 89% of the population in Nepal currently has access to at-least basic water supply services and 62% to basic sanitation facilities, providing safe water quality at the point of consumption and ensuring adequate hygiene practices remain challenges [14, 15]. In a recent study, 31.5% of children in the Eastern region of Nepal were found to be infected with intestinal parasites. Parasitic infections were significantly associated with not using soap after defecation, the habit of thumb sucking, and not wearing sandals [16]. However, the health and nutritional status of children and their associations with nutrition and WASH have not been extensively investigated in remote hilly areas of Nepal. The Demographic and Health Survey (DHS) 2016 showed that 1 in 25 children in Nepal dies before reaching the age of 5 years, and almost 3500 die yearly from preventable causes [17, 18]. Diarrhoea is one of the most common illnesses among children and continues to be a major cause of childhood morbidity and mortality [17]. However, efforts to combat health and nutritional problems among children in these settings do not effectively incorporate WASH interventions. Hence, the aim of this study was to assess the influence of nutrition practices and WASH infrastructure on the nutritional and health status of children aged 6 months to 10 years in three rural hilly areas of Nepal. The findings from this study provide a crucial benchmark for delivering subsequent public health interventions.

## Methods

### Study area

The survey area was located in the districts of Surkhet (A and B), Achham, and Dailekh in the Karnali province of Nepal. The sites were selected according to the following criteria: (a) mountainous region with extremely remote location, (b) availability of a piped water supply scheme in communities WARM-P training (i.e. hygiene education) has not taken place, and (c) the population not having access to products for household water treatment.

### Study design, sample population, sample size and sampling methods

This cross-sectional study was conducted from March to May 2018 and involved 1427 households with children aged 6 months to 10 years. Sample size and statistical power were calculated using G*Power 3.1. A sample size of 300 households was required at each of the four sites to detect an effect in Cohen’s f2 at one-tailed alpha of 0.05 and a statistical power of 90% with mixed logistic regression and 15 predictor variables adjusting for clustering effect of the study site [19, 20]. We therefore randomly sampled a minimum of 345 households at each of the four sites.

### Questionnaire survey

A quantitative, structured questionnaire was administered to the children’s caregivers (mostly mothers; Supplementary file 1). The questionnaire was developed following international guidelines and there were both closed and open-ended questions developed from standardized questions following international guidelines [21, 22]. The questionnaire was coded in Open Data Kit software on tablets (Samsung Galaxy note 10.1 N8010) [23] and contained questions on the use of water sources, psychological factors concerning water handling and hygiene practices, observations of WASH infrastructure, information on WASH promotion activities received, nutrition provided to children, and children’s history of waterborne illness in the past 7 days. The interviews were complemented by structured observations and the questionnaires were pretested and adapted to meet the conditions of the study site [24].

### Child diet, household food security and signs of nutritional deficiency

Child dietary information was assessed following the guidelines of the Food and Agricultural Organisation (FAO) [22]. The caregivers were requested to recall whether nine different food groups were consumed within the past 7 days and, if consumed, the frequency of consumption. The supervisors randomly re-interviewed a subset of 10% of the surveyed households to assess reproducibility. Household food security was assessed with questions relating to the availability of food during the entire year.

Certified medical assistants screened children for the clinical signs of nutritional deficiencies using a standard checklist. They were examined for; (a) wasted appearance, (b) loss of hair pigment and easy pluckability, (c) bitot’s spots, (d) dry and infected cornea, (e) oedema, (f) several types of dermatitis, (g) spongy bleeding gums, (h) pale conjunctiva, (i) red inflamed tongue, (j) sub-dermal haemorrhage, (k) bowed legs, and (l) goitre [25].

### Anthropometric measurements

The children were examined for anthropometric measurements; height or length and weight, adhering to standard procedures [26]. Supine lengths were obtained from children younger than 2 years using Seca BabyMat 210 and for children aged between 24 months and 10 years, using a height-measuring board and a digital scale (Seca 877; Hamburg, Germany) [24]. Anthropometric indices were calculated using AnthroPlus (WHO; Geneva, Switzerland) in accordance with the World Health Organisation (WHO) guidelines [26, 27]. Three anthropometric indices were expressed as z-scores (i.e. differences from the median in standard deviations): (a) weight for age (WAZ, underweight), (b) height for age (HAZ, stunting), and (c) body mass index for age (BMIZ, thinness) [4]. Z-scores of ≥ − 2 were regarded as normal, those between < − 2 and ≥ − 3 as moderate undernutrition and those below <− 3 as severe undernutrition. Children were considered to be undernourished if at least one of the anthropometric indices indicated undernutrition [26, 27].

### Parasitological survey

Caregivers were asked to provide a fresh morning stool sample without urine contamination from the participating child on the day following the household survey. The samples were processed on the same day by experienced laboratory technicians. Each stool sample was analysed using direct wet-mount and formalin-ether concentration techniques following standard guidelines [28–30]. In addition, a duplicate Kato-Katz thick smear was prepared for the diagnosis of helminths [31]. The presence of infection by any worm species was defined by the detection of one or more eggs on either slide [7]. The infection intensity of helminths was calculated according to criteria defined by the WHO and multiplied by 24 to reach the total number of eggs per gram (EPG) of stool [5, 32]. Stool samples were obtained from 962 children.

### Drinking water quality examination

Water samples were collected from the household’s main drinking water source and from the container used for drinking water transport and storage. The sample at the source was taken after letting the water run for 60 s from the tap. Caregivers were requested to bring fresh drinking water from the source to the household in the same container they usually use for this [33]. Water samples for analysis were poured into sterile Nasco Whirl Pak bags and immediately analysed using the membrane filtration technique: 100 mL water samples were passed through sterile 0.45 μm millipore cellulose membrane filters with sterilized filtration equipment. The filter pads were plated on Nissui Compact Dry Coli-scan plates and incubated for 24 h at 35 +/− 2 °C. Colony-forming units of total coliforms and *Escherichia coli* (*E. coli*) were counted after 24 h of incubation [15].

### Data management and statistical analysis

Data cleaning was performed daily, and if any values were missing or inconsistent, the respective household was consulted the following day. Readings of intestinal parasite and nutritional deficiency screenings were double entered into an Excel 2010 spread sheet (Microsoft; Redmond, USA) and cross-checked. Numerical variables were described by means and standard deviations if normally distributed and by medians and interquartile range otherwise. Categorical variables were described by absolute and relative frequencies. We employed χ^*2*^ statistics to assess the differences in distribution of categorical variables between the study areas. Household socioeconomic status was characterized based on factor analysis of reported household assets. Three factors reflecting three socioeconomic domains were retained and divided using the *k*-means procedure into three categories; (a) low, (b) medium, and (c) high [34]. The same procedure was applied to create one variable for the cleanliness of containers used for transport and storage of drinking water, latrine hygiene, cleanliness of the household environment and kitchen, and personal hygiene. For each of these variables, three factors were retained and categorized, indicating (a) low, (b) intermediate, and (c) high categories.

We assessed four health-related outcome variables: (a) undernutrition (i.e. stunting, underweight and thinness) (b) nutritional deficiencies (c) intestinal parasitic infection and (d) diarrhoea. Since only a few undernutrition cases were severe, the cases were pooled into a binary variable of stunted/non-stunted, and underweight/non-underweight for the subsequent analysis. Similarly, there was a low prevalence of parasites, such as *T. trichiura*, *E. vermicularis* and *Ancylostoma duodenale*. Therefore, all reported intestinal parasitic infections were pooled into a binary variable of parasite infection/no infection to maximize statistical power. Nutritional deficiencies and diarrhoea outcomes were coded into binary variables for the subsequent comparative analysis.

We assessed associations between the binary outcome variables and hypothesized risk factors using mixed logistic regression models with random intercepts of study sites, controlling for potential confounding by age, sex, and socioeconomic status. First, the associations between outcome variables and risk factors were assessed using univariate models. Variables with *P*-values < 0.2 were retained for the final model [35]. Odds ratios were reported and the associations were considered as statistically significant if *P*-values were < 0.05. The statistical analysis was performed with STATA version 14 (STATA Corporation, College Station, TX, USA).

## Results

### Socio-demographic characteristics of the study participants

The socio-demographic and socioeconomic characteristics of the interviewed households are provided in Table A in the supplementary materials. Caregivers aged 25–39 years constituted the largest group (57.9%) of interviewees. More than 80% of the caregivers could both read and write. Agriculture was the main (60.6%) occupation of the household heads. The majority of children (99.1%) included in the study were between 6 months and 5 years of age, while 0.9% were between 6 to 10 years of age. 59.7% of the households kept animals inside the home overnight and the majority (84.1%) of the households had mud floors. Around 52.7% of the households across the study sites had access to electricity.

### Child-feeding practices and household food security

Almost all caregivers (99.6%) reported having breastfed the participating child until the age of 6 months. The dietary diversity scores were low with 11.2% of the households having consumed all nine listed food groups in the previous 7 days of the survey (Table B and Table C, supplementary materials). The consumption of milk/milk products and eggs at least once per week was 9.2 and 5.3%, respectively (Table C, supplementary materials). About 40% of the households did produce their own food, among which 20.8% reported self-sufficient yearly food production.

### Prevalence of nutritional deficiencies and associated risk factors

A total of 63.9% of the children in the study suffered from at least one sign of a nutritional deficiency. About one third (35.9%) of the children suffered from pale conjunctiva, followed by Bitot’s spots (19.8%), red inflamed tongue (18.3%), spongy bleeding gums (16.3%), wasted appearance (13.8%), dry and infected cornea (13.2%), loss of hair pigment (10.7%), sub-dermal haemorrhage (4.6%), oedema (2.7%), bowed legs (2.6%), and goitre (0.6%) (Table 1).

**Table 1:** Prevalence of children’s undernutrition and signs of nutritional deficiencies in four study sites

Malnutrition indicators, clinical outcomes and nutritional deficiency	[N (%)]	Sex		*P-value*	Age group		*P-value*	Area				P-value*
		Male	Female		< 5 years	> 5 years		Surkhet A [n (%)]	Surkhet B [n (%)]	Dailekh [n (%)]	Accham [n (%)]	
Malnutrition indicators												
Stunting (*n* = 1389)												
Severe^a^	248 (17.9)	146 (18.8)	102 (16.7)	0.51	158 (17.8)	90 (17.9)	0.81	29 (8.5)	49 (13.8)	87 (25.1)	83 (23.9)	**0.01**
Moderate^b^	370 (26.6)	209 (26.9)	161 (26.3)		241 (27.2)	129 (25.6)		73 (21.5)	83 (23.3)	104 (30.1)	110 (31.7)	
Normal^c^	771 (55.5)	422 (54.3)	349 (57.0)		487 (55.0)	284 (56.5)		238 (70.0)	224 (62.9)	155 (44.8)	154 (44.4)	
Thinness (*n* = 1344)												
Severe^a^	55 (4.1)	25 (3.4)	30 (5.0)	0.31	36 (4.2)	19 (3.9)	0.83	12 (3.7)	14 (3.9)	16 (4.8)	13 (4.0)	0.31
Moderate^b^	96 (7.1)	54 (7.2)	42 (7.0)		63 (7.4)	33 (6.7)		19 (5.9)	22 (6.2)	34 (10.2)	21 (6.4)	
Normal^c^	1193 (88.8)	667 (89.4)	526 (88.0)		752 (88.4)	441 (89.4)		293 (90.4)	322 (89.9)	284 (85.0)	294 (89.6)	
Underweight (*n* = 1360)												
Severe^a^	142 (10.4)	83 (11.0)	59 (9.8)	0.63	88 (10.2)	54 (10.9)	0.88	16 (4.9)	33 (9.2)	49 (14.4)	44 (13.3)	**0.01**
Moderate^b^	265 (19.5)	151 (20.0)	114 (18.9)		171 (19.8)	94 (19.0)		40 (21.1)	57 (15.8)	85 (25.1)	83 (25.1)	
Normal^c^	953 (70.1)	522 (69.0)	431 (71.3)		606 (70.1)	347 (70.1)		274 (83.0)	270 (75.0)	205 (60.5)	204 (61.6)	
Clinical outcomes and signs/symptoms of nutritional deficiency (*n* = 1427)												
Wasted appearance	197 (13.8)	95 (12.0)	102 (16.0)	**0.03**	145 (16.0)	52 (10.0)	**0.01**	29 (8.3)	33 (9.0)	66 (18.5)	69 (19.3)	**0.01**
Bitot’s spot	283 (19.8)	168 (21.3)	115 (18.1)	0.13	147 (16.2)	136 (26.2)	**0.01**	27 (7.8)	65 (17.8)	89 (25.0)	102 (28.5)	**0.01**
Loss of hair pigment	153 (10.7)	80 (10.1)	73 (11.5)	0.42	114 (12.6)	39 (7.5)	**0.01**	12 (3.5)	46 (12.6)	56 (15.7)	39 (10.9)	**0.01**
Dry and infected cornea	189 (13.2)	108 (13.7)	81 (12.7)	0.60	114 (12.6)	75 (14.5)	0.31	25 (7.2)	26 (7.1)	49 (13.8)	89 (24.9)	**0.01**
Oedema	38 (2.7)	18 (2.3)	20 (3.14)	0.32	28 (3.1)	10 (1.9)	0.91	4 (1.2)	5 (1.4)	9 (2.5)	20 (5.6)	**0.01**
Pale conjunctiva	513 (35.9)	279 (35.3)	234 (36.7)	**0.01**	309 (34.0)	204 (39.3)	**0.05**	83 (23.9)	95 (26.0)	154 (43.3)	181 (50.6)	**0.01**
Bowed legs	37 (2.6)	25 (3.2)	12 (1.9)	0.13	23 (2.5)	14 (2.7)	0.85	7 (2.0)	3 (0.8)	9 (2.5)	18 (5.0)	**0.01**
Spongy bleeding gums	232 (16.3)	131 (16.6)	101 (15.9)	0.71	104 (11.5)	128 (24.7)	**0.01**	37 (10.6)	68 (18.6)	70 (19.7)	57 (15.9)	**0.01**
Dermatitis	818 (57.3)	464 (58.7)	354 (55.8)	0.23	511 (56.3)	307 (59.2)	0.29	155 (44.5)	200 (54.8)	235 (66.0)	228 (63.7)	**0.01**
Red inflamed tongue	261 (18.3)	138 (17.5)	123 (19.3)	0.37	167 (18.4)	94 (18.1)	0.90	24 (6.9)	62 (17.0)	80 (22.5)	95 (26.5)	**0.01**
Sub-dermal haemorrhage	66 (4.6)	39 (4.9)	27 (4.2)	0.53	36 (4.0)	30 (5.8)	0.12	8 (2.3)	16 (4.4)	19 (5.3)	23 (6.4)	**0.06**
Goitre	9 (0.63)	7 (0.9)	2 (0.3)	0.18	7 (0.8)	2 (0.4)	0.38	1 (0.3)	3 (0.8)	1 (0.3)	4 (1.1)	0.41
Overall nutritional deficiency (N = 1427)	1113 (78.0)	58 (72.5)	1055 (78.3)	0.22	697 (76.8)	416 (80.2)	0.14	216 (62.1)	269 (73.7)	306 (86.0)	322 (89.9)	**0.01**

^a^ z score of < −3

^b^ z score of < −2 and ≥ −3

^c^ z score ≥ −2

*P-values are calculated by χ2 test

Children > 5 years old had twice the odds, all else constant, of having nutritional deficiencies compared to their younger counterparts (AOR = 1.84; 95% CI: 1.30–2.62; *P* = 0.01). Children whose caregivers washed their hands after cleaning a baby’s bottom were at lower odds of having nutritional deficiencies (AOR = 0.60; 95% CI = 0.40–0.92; *P* = 0.02) compared to children whose caregivers did not follow such practice. Children living in houses where animals were kept inside overnight had 1.71 times higher odds of having signs of nutritional deficiencies (AOR = 1.71; 95% CI = 1.17–2.51; *P* = 0.01) than their counterparts. Children from households producing their own food were significantly better protected against nutritional deficiencies (AOR = 0.51; 95% CI: 0.35–0.76; *P* = 0.01) than were children without their own food production. Children from households in the category of high latrine hygiene were at lower odds of nutritional deficiencies (AOR = 0.61; 95% CI: 0.41–0.91; *P* < 0.001) than those living in households with low latrine hygiene. Similarly, a high level of kitchen hygiene decreased children’s odds of nutritional deficiencies compared to low kitchen hygiene (AOR = 0.4; 95% CI 0.22–2.76; *P* = 0.008). Being in the intermediate or lower category of personal hygiene increased a child’s odds for clinical signs of nutritional deficiencies by 1.84 and being in the higher category by 1.9 (AOR = 1.84; 95% CI: 1.22–2.76; *P* = 0.005 and AOR = 1.9; 95% CI: 1.17–3.1; *P* = 0.005). Children from households with coliform bacteria in their drinking water sources had 10.4 times higher odds of having symptoms of nutritional deficiencies (AOR = 10.4; 95% CI: 1.61–67.42; *P* = 0.01) than children from households with an uncontaminated water source (Table 2).

**Table 2:** Association between nutritional deficiencies^a^ and various factors in univariate and multivariate logistic regression analyses

Nutritional deficiencies [N(cases)=912]		Univariate logistic regression^b^			Multivariate logistic regression^c^		
		OR	95% CI	*P-value*	aOR	95% CI	*P-value*
Age of the participating child	< 5 years	1.00			1.00		
	> 5 years	1.59	1.25-2.02	**0.01**	1.84	1.30-2.62	**0.01**
Sex of the participating child	Male	1.00			1.00		
	Female	0.98	0.78-1.23	0.84	1.04	0.76-1.42	0.80
Number of children in the household	< 5	1.00			1.00		
	> 5	2.81	0.60-13.27		3.05	0.32-29.12	0.33
Socioeconomic status^d^	Poor	1.00			1.00		
	Intermediate	1.14	0.83-1.58	0.08	1.10	0.71-1.72	0.43
	High	1.20	0.85-1.71		1.38	0.82-2.33	
Occupation of the household head	Agriculture	0.96	0.76-1.21	0.73			
	Business	0.75	0.52-1.09	**0.13**	0.84	0.49-1.45	0.54
	Daily labourer	0.65	0.51-0.83	**0.01**	0.75	0.52-1.06	0.10
	Government service	1.04	0.59-1.83	0.88			
	Other independent work	0.25	0.04-1.36	**0.11**	0.51	0.07-3.67	0.50
	None	4.93	0.62-39.22	**0.13**	2.70	0.22-32.85	0.44
Handwashing with soap	<5 times	1.00			1.00		
	5-10 times	0.66	0.51-0.85	**0.007**	1.08	0.65-1.80	0.53
	>10 times	0.65	0.12-3.44		3.39	0.36-31.77	
Times of handwashing	When they look dirty	0.56	0.44-0.71	**0.01**	0.74	0.50-1.08	0.12
	After going to toilet	0.39	0.14-1.09	**0.07**	0.13	0.01-1.10	0.06
	After cleaning baby’s bottom	0.40	0.32-0.52	**0.01**	0.60	0.40-0.92	**0.02**
	Before eating	1.02	0.78-1.33	**0.15**	0.75	0.49-1.15	0.19
	Before cooking	1.44	1.14-1.82	**0.01**	1.07	0.67-1.68	0.78
Animals inside home overnight	"yes" *vs.* "no"	1.67	1.32-2.13	**0.01**	1.71	1.17-2.51	**0.01**
Information received on WASH^e^	"yes" *vs.* "no"	0.65	0.45-0.95	**0.02**	1.03	0.56-1.89	0.93
Child suffered from any illnesses:							
Fever	"yes" *vs.* "no"	0.98	0.78-1.24	0.89			
Cough	"yes" *vs.* "no"	1.09	0.86-1.37	0.47			
Respiratory difficulties	"yes" *vs.* "no"	1.97	1.40-2.78	**0.01**	1.32	0.79-2.18	0.29
Diarrhea^f^	"yes" *vs.* "no"	1.05	0.77-1.44	0.75			
Blood in stool	"yes" *vs.* "no"	2.87	1.25-6.60	**0.01**	1.72	0.50-5.90	0.39
Mucus in stool	"yes" *vs.* "no"	3.10	1.42-6.75	**0.01**	2.07	0.67-6.38	0.20
Blood in urine	"yes" *vs.* "no"	1.99	0.39-9.98	0.40			
Heard about intestinal parasites^g^	"yes" *vs.* "no"	0.53	0.40-0.70	**0.01**	1.12	0.46-2.69	0.80
Awareness on measures against intestinal parasites	Washing hands with soap	0.39	0.27-0.57	**0.01**	0.56	0.26-1.24	0.15
	Cutting finger nails	0.22	0.13-0.37	**0.01**	0.83	0.32-2.15	0.71
	Wash fruits/vegetables before consumption	0.13	0.05-0.33	**0.01**	0.64	0.17-2.45	0.52
	Wear shoe	0.13	0.07-0.23	**0.01**	0.24	0.09-0.62	**0.01**
	Drink clean water	0.61	0.45-0.82	**0.01**	1.63	0.67-3.95	0.28
	Deworming regularly	0.85	0.60-1.21	0.38			
Child ever breastfed	“yes” vs. “no”	0.80	0.12-5.17	0.82			
Total months child breastfed	<6 months	2.82	1.07-7.42		3.99	0.86-18.50	
	6-12 months	1.64	0.62-4.33	<0.001	1.90	0.41-8.84	**<0.001**
	>12 months	1.00			1.00		
Complementary feeding of the participating child started <6months	"yes" *vs.* "no"	0.57	0.30-1.09	**0.09**	0.52	0.17-1.62	0.26
Material of the home’s floor	"earth" vs. "cement"	1.01	0.74-1.37	0.95			
Dietary diversity scores	1	1.00			1.00		
	2	1.07	0.66-1.74		0.75	0.36-1.54	
	3	1.15	0.72-1.85		0.70	0.34-1.46	
	4	2.06	1.23-3.44		1.12	0.52-2.44	
	5	1.86	1.09-3.12	**<0.001**	1.67	0.77-3.61	0.08
	6	1.28	0.78-2.10		0.89	0.43-1.84	
	7	1.30	0.79-2.16		0.93	0.44-1.96	
	8	0.83	0.51-1.37		0.84	0.41-1.74	
	9	0.27	0.17-0.45		0.51	0.25-1.05	
Production of own food	"yes" *vs.* "no"	0.52	0.41-0.67	**0.01**	0.51	0.35-0.76	**0.01**
Latrine hygiene	Lower category	1.00			1.00		
	Intermediate category	1.38	1.02-1.86	**<0.001**	1.43	0.93-2.20	**<0.001**
	High category	0.68	0.52-0.90		0.61	0.41-0.91	
Kitchen hygiene	Lower category	1.00			1.00		
	Intermediate category	1.33	1.01-1.74	**<0.001**	0.89	0.58-1.34	**0.008**
	High category	0.47	0.34-0.65		0.40	0.22-0.75	
Personal hygiene of participating child and their caregivers	Lower category	2.24	1.68-3.00		1.90	1.17-3.10	
	Intermediate category	1.93	1.46-2.55	**<0.001**	1.84	1.22-2.76	**0.005**
	High category	1.00			1.00		
Hygiene status of water transport container	Lower category	1.00			1.00		
	Intermediate category	0.97	0.68-1.38	**<0.001**	0.60	0.23-1.58	0.58
	High category	0.55	0.42-0.72		0.91	0.31-2.66	
Hygiene status of water storage container	Lower category	1.00			1.00		
	Intermediate category	1.10	0.77-1.59	**<0.001**	1.84	0.69-4.87	0.33
	High category	0.54	0.42-0.72		0.77	0.26-2.25	
Presence of intestinal parasites	"yes" *vs.* "no"	0.88	0.66-1.17	0.37			
Presence of undernutrition	"yes" *vs.* "no"	1.07	0.84-1.35	0.59			
*E. coli* in POC^h^ drinking water	"yes" *vs.* "no"	0.93	0.55-1.57	0.79			
Coliforms in POC drinking water	"yes" *vs.* "no"	6.53	1.24-34.29	**0.03**	10.44	1.61-67.4	**0.01**
*E. coli* in POU^i^ drinking water	"yes" *vs.* "no"	1.08	0.63-1.85	0.78			
Coliforms in POU drinking water	"yes" *vs.* "no"	2.33	0.79-6.88	**0.13**	1.78	0.33-9.46	0.50

^a^Nutritional deficiency: presence of wasted appearance, bitot’s spot, loss of hair pigment, dry and infected cornea, oedema, pale conjunctiva, bowed legs, spongy bleeding gums, dermatitis, red inflamed tongue, sub-dermal haemorrhage and goitre

^b^Odds ratios were obtained from univariate mixed logistic regression models with random area intercepts, and *P*-values were obtained from Wald- and likelihood ratio tests. *P*-values <0.2 in the univariate analyses are marked in bold

^c^Adjusted odds ratios were obtained from a multivariable mixed logistic regression model with random area intercepts including all variables with *P*-values < 0.2 in the univariate models along with gender and age group of the child and socio-economic category of the household. *P*-values were obtained from Wald- and likelihood ratio tests and values < 0.05 are marked in bold

^d^Socio-economic status was derived from a factor analysis using principal component analysis of variables indicating the wealth index

^e^Water, sanitation and hygiene

^f^Passage of loose stool three or more than three times per day

^g^Overall soil transmitted helminths and intestinal protozoa

^h^Point of collection

^I^Point of use

### Prevalence of undernutrition and associated risk factors

Table 1 shows the percentage distribution of undernutrition in the study sample by sex, age group, and study sites. The prevalence of undernutrition was 55.5%, while the prevalence of stunting was 44.5%, thinness 11.2%, and underweight 29.9%.

Table 3 provides an overview of the association between undernutrition and associated risk factors in univariate and multivariate regression analysis. A higher and intermediate socioeconomic status had a significant negative association with undernutrition with odds ratios of 0.70 (95% CI: 0.43–1.11) and 0.43 (95% CI: 0.25–0.75), respectively. Children of caregivers with sound knowledge about the importance of regular deworming had lower odds of undernutrition (AOR = 0.44; 95% CI = 0.20–0.94; *P* = 0.03) than children having caregivers lacking such knowledge. Children receiving supplementary food were at significantly lower odds of being undernourished (AOR = 0.57; 95% CI: 0.38–0.84; *P* = 0.01) than children without supplementary food. Similarly, children from households producing their own food were at significantly lower odds of being undernourished (AOR = 0.67; 95% CI: 0.46–0.97; *P* = 0.03) than those from households without agricultural production.

**Table 3:** Association of undernutrition^a^ with various factors in univariate and multivariate logistic regression analysis

Undernutrition [N (cases)=760]		Univariate logistic regression^b^			Multivariate logistic regression^c^		
		OR	95% CI	*P-value*	aOR	95% CI	*P-value*
Age of the participating child	< 5 years	1.00			1.00		
	> 5 years	0.98	0.78-1.23	0.83	1.01	0.72-1.42	0.95
Sex of the participating child	Male	1.00			1.00		
	Female	1.01	0.81-1.27	0.89	0.95	0.68-1.33	0.77
Number of children in the household	< 5	1.00					
	> 5	1.05	0.32-3.42	0.93			
Socioeconomic status	Poor	1.00			1.00		
	Intermediate	0.73	0.54-0.99	**0.006**	0.70	0.43-1.11	**0.01**
	High	0.57	0.41-0.80		0.43	0.25-0.75	
Caregivers’ literacy	Can neither read or write	0.75	0.30-1.88				
	Can read only	1.10	0.80-1.52	0.60			
	Can both read and write	1.00					
Occupation of the household head	Agriculture	1.22	0.96-1.53	**0.10**	1.05	0.71-1.53	0.82
	Business	0.90	0.62-1.29	0.55			
	Daily labourer	1.11	0.88-1.40	0.37			
	Government service	0.50	0.28-0.92	**0.03**	0.54	0.21-1.38	0.20
	Other independent work	0.46	0.46-13.08	0.29			
	None	0.38	0.11-1.32	**0.13**	0.49	0.12-2.04	0.33
Household involved in management of the water system	"yes" *vs.* "no"	0.84	0.51-1.40	0.51			
Handwashing with soap	<5 times	1.00					
	5-10 times	0.93	0.72-1.20	0.85			
	>10 times	1.04	0.21-5.29				
Animals inside home overnight	"no" *vs.* "yes"	0.94	0.74-1.20	0.64			
Information received on WASH	"yes" *vs.* "no"	0.91	0.71-1.18	0.49			
Heard about intestinal parasites	"yes" *vs*. "no"	0.77	0.59-1.01	**0.06**	1.39	0.76-2.55	0.29
Awareness on measures against intestinal parasites	Wash hands with soap	0.92	0.64-1.31	0.65			
	Drink clean water	0.84	0.63-1.13	0.26			
	Regular deworming	0.65	0.46-0.92	**0.02**	0.44	0.20-0.94	**0.03**
Complementary feeding of the participating child started <6months	"yes" *vs*. "no"	0.64	0.33-1.23	**0.18**	1.95	0.60-6.36	0.27
Received additional meal (snacks)	"yes" *vs*. "no"	0.65	0.51-0.83	**<0.001**	0.57	0.38-0.84	**0.01**
DDS^d^	1	1.00			1.00		
	2	1.09	0.67-1.77		1.01	0.47-2.15	
	3	1.41	0.89-2.25		0.85	0.41-1.74	
	4	1.13	0.71-1.83		0.90	0.44-1.84	
	5	1.35	0.84-2.18	0.16	0.84	0.40-1.75	**0.03**
	6	1.02	0.64-1.64		0.50	0.24-1.04	
	7	1.85	1.13-3.03		1.69	0.77-3.73	
	8	1.26	0.78-2.05		0.84	0.40-1.77	
	9	1.61	0.99-2.61		1.76	0.80-3.90	
Production of own food	"yes" *vs*. "no"	0.86	0.68-1.07	**0.18**	0.67	0.46-0.97	**0.03**
*Giardia lamblia*	"yes" *vs*. "no"	0.91	0.64-1.28	0.57			
Presence of intestinal helminths	"yes" *vs*. "no"	1.27	0.94-1.70	**0.12**	1.36	0.93-1.98	0.11
*Ascaris lumbricoides*	"yes" *vs*. "no"	1.15	0.82-1.62	0.41			
*Trichuris trichiura*	"yes" *vs*. "no"	1.68	0.31-9.19	0.55			
*Hymenolepsis nana*	"yes" *vs*. "no"	1.48	0.75-2.93	0.26			
*Enterobius vermicularis*	"yes" *vs*. "no"	2.19	0.85-5.62	**0.10**	2.01	0.70-5.79	0.19
Latrine hygiene	Lower category	1.00					
	Intermediate category	1.11	0.85-1.45	0.64			
	High category	1.12	0.85-1.47				
Kitchen hygiene	Lower category	1.27	0.98-1.65		1.26	0.82-1.94	
	Intermediate category	1.15	0.84-1.58	0.21	0.86	0.48-1.53	0.23
	High category	1.00			1.00		
Personal hygiene of participating child and their caregivers	Lower category	1.32	1.01-1.73		1.43	0.92-2.22	
	Intermediate category	1.04	0.79-1.37	0.10	0.83	0.51-1.35	**0.05**
	High category	1.00			1.00		
*E. coli* at POU^e^ drinking water	"yes" *vs*. "no"	1.48	0.84-2.59	**0.17**	0.87	0.34-2.24	0.77
E. coli at POC^f^ drinking water	"yes" *vs*. "no"	1.63	0.96-2.76	**0.07**	1.27	0.59-2.71	0.25
Total coliforms at POC drinking water	"yes" *vs*. "no"	5.31	1.01-27.9	**0.05**	3.81	0.32-45.49	0.29
Total coliforms at POU drinking water	"yes" *vs*. "no"	2.32	0.72-7.48	**0.16**	1.47	0.18-12.04	0.72
Presence of diarrhoea	"yes" *vs*. "no"	1.08	0.81-1.46	0.59			
Presence of nutritional deficiencies	"yes" *vs*. "no"	1.06	0.84-1.35	0.63			

^a^Undernutrition included the presence or absence of stunting, BMI Z(thinness) or unerweight

^b^Odds ratios were obtained from univariate mixed logistic regression models with random area intercepts, and *P*-values were obtained from Wald- and likelihood ratio tests. *P*-values <0.2 in the univariate analyses are marked in bold

^c^Adjusted odds ratios were obtained from a multivariate mixed logistic regression model with random area intercepts including all variables with *P*-values < 0.2 in the univariate models along with gender and age group of the child and socio-economic category of the household. *P*-values were obtained from Wald- and likelihood ratio tests and values < 0.05 are marked in bold

^d^Dietary diversity score

^e^Point of use

^f^Point of collection

### Prevalence of intestinal parasites and associated risk factors

Tables 4 and 5 show the prevalence of intestinal parasitic infections in the study population and associated risk factors. The overall prevalence of intestinal parasitic infection is 51.1%. The predominant helminth species infecting the children were *Ascaris lumbricoides* (21.1%), followed by *Hymenolepsis nana* (4.6%), *Ancylostoma duodenale* (3.2%), *Enterobius vermicularis* (2.7%), and *Trichuris trichiura* (0.7%). Polyparasitism and co-infection were not common. About 23.4% of the children were infected with *Giardia intestinalis*.

**Table 4:** Prevalence and intensity of intestinal helminths and protozoa infections among children [*N* = 962]

Parasite (No. of samples examined =962)	Prevalence of intestinal parasites [n (%)]	Sex		*P-value*	Age group		*P-value*	Study Site				*P-value*	Mean eggs per gram±SE (epg^b^)
		Male	Female		< 5 years	> 5 years		Surkhet A [n(%)]	Surkhet B [n (%)]	Dailekh [n (%)]	Accham [n (%)]		
Nematodes													
*Trichuris trichiura*^*a*^	7 (0.7)	4 (0.7)	3 (0.7)	0.99	1 (0.2)	6 (1.6)	0.01	2 (0.8)	0 (0.0)	3 (1.7)	2 (0.8)	0.23	4.15 ± 43.0
Hookworm^a^	31 (3.2)	16 (2.9)	15 (3.6)	0.54	18 (3.0)	13 (3.6)	0.64	13 (5.0)	7 (2.5)	8 (4.5)	3 (1.2)	0.06	16.8 ± 92.6
*Enterobius vermicularis*^*a*^	26 (2.7)	15 (2.7)	11 (2.7)	0.94	14 (2.4)	12 (3.3)	0.38	3 (1.2)	4 (1.4)	9 (5.1)	10 (4.0)	**0.02**	20.8 ± 99.4
*Ascaris lumbricoides*^*a*^	203 (21.1)	114(20.8)	89(21.5)	0.79	140(23.5)	63(17.3)	0.02	46(17.8)	68 (24.5)	68(38.4)	21 (8.4)	**0.01**	110 ± 238.4
Cestodes													
*Hymenolepsis nana*^*a*^	44 (4.6)	18 (3.3)	26 (6.3)	**0.03**	27 (4.5)	17 (4.7)	0.92	1 (0.4)	9 (3.2)	10 (5.7)	24 (9.6)	**0.01**	34.4 ± 152.2
Intestinal protozoa													
*Giardia lamblia*	225 (23.4)	144 (26.3)	81 (19.6)	**0.02**	145 (24.3)	80 (21.9)	0.40	67 (26.0)	12 (4.3)	33 (18.6)	113 (45.4)	**0.01**	

^a^ The intensity of intestinal helminths in all participating children is light infection i.e. for *Trichuris trichiura*: 1–999; hookworm: 1–1999; *Enterobius vermicularis*: 1–2999; *Ascaris lumbricoides*: 1–4999

^b^ Egg counts/ egg per gram of faeces describe the intensity of parasitic infection

**Table 5:** Association of factors with parasitic infections in univariate and multivariate logistic regression

Overall parasitic infection [N (total) =962/ N (cases)=492]		Univariate logistic regression^a^			Multivariate logistic regression^b^		
		OR	95% CI	*P-value*	aOR	95% CI	*P-value*
Age of the child	< 5 years	1.00			1.00		
	> 5 years	0.86	0.66-1.13	0.29	0.86	0.65-1.15	0.31
Sex of the child	Male	1.00			1.00		
	Female	0.90	0.69-1.17	0.43	0.88	0.66-1.16	0.37
Number of children in the household	< 5	1.00					
	> 5	2.01	0.49-8.11	0.34			
Caregivers can read/write	Can neither read or write	1.09	0.74-1.61		1.18	0.78-1.80	
	Can read only	3.15	0.83-12.0	**0.18**	4.19	1.03-17.0	0.08
	Can both read and write	1.00			1.00		
Involvement in the water supply system in the community	"no" *vs.* "yes"	1.89	1.05-3.42	**0.03**	1.60	0.84-3.05	0.15
Socioeconomic status	Poor	1.00			1.00		
	Middle	0.88	0.60-1.30	0.27	0.87	0.58-1.31	0.45
	Better	1.16	0.75-1.78		1.09	0.68-1.75	
Main drinking water source	Piped water in the house	1.00					
	Piped water in the village	1.39	0.87-2.21				
	Open source	1.33	0.47-3.73	0.29			
	Protected source	1.18	0.40-3.49				
	River, stream or canal	6.79	0.72-64.33				
Time to fetch drinking water	< 5 minutes	1.00					
	5-15 minutes	1.53	0.66-3.55	0.40			
	16-60 minutes	1.23	0.53-2.78				
	> 60 minutes	1.07	0.38-3.01				
Main drinking water source functioning now	Functioning well	1.00					
	Functioning irregularly	1.24	0.61-1.96	0.76			
Interruption of main drinking water source for more than a week	"no" *vs.* "yes"	1.76	0.91-3.41	**0.09**	1.30	0.64-2.64	0.47
Knowledge on factors that make water unsafe for drinking	Open unprotected source	1.09	0.77-1.54	0.62			
	Open defecation	1.09	0.83-1.45	0.53			
	Deforestation	1.97	0.97-3.99	**0.06**	1.84	0.87-3.89	0.11
Method of drinking water treatment used	Boiling	0.96	0.50-1.86	0.91			
	Filtration with cloth	0.77	0.33-1.85	0.57			
	Use of filter ("yes" *vs.* "no")	1.55	1.00-2.41	**0.05**	1.27	0.78-2.09	0.34
Clean drinking water storage container	"no" *vs.* "yes"	1.88	1.04-3.41	**0.04**	1.65	0.87-3.17	0.13
Handwashing with soap	<5 times	1.00					
	5-10 times	1.08	0.79-1.47	0.32			
	>10 times	0.25	0.03-2.23				
Times of handwashing	When they look dirty	0.10	0.76-1.31	0.99			
	After going to toilet	0.58	0.23-1.49	0.26			
	After cleaning baby’s bottom	1.00	0.77-1.31	0.98			
	Before eating	1.07	0.79-1.46	0.65			
	Before cooking	1.14	0.87-1.48	0.35			
Animals inside home overnight	"yes" *vs.* "no"	1.13	0.84-1.51	0.41			
Information received on WASH^c^	"no" *vs.* "yes"	1.90	1.21-3.00	**0.01**	1.10	0.44-2.72	0.84
Handwashing station installed	"no" *vs.* "yes"	2.66	1.05-6.73	**0.04**	1.66	0.54-5.17	0.38
Use soap always to wash hands	"yes" *vs.* "no"	1.69	0.96-2.99	**0.07**	1.22	0.48-3.10	0.67
Wash hands during critical times	"yes" *vs.* "no"	1.98	0.97-3.67	**0.06**	0.73	0.27-1.95	0.53
Sometimes treating water	"no" *vs.* "yes"	3.68	1.39-9.71	**0.01**	2.29	0.72-7.23	0.16
Attended hygiene literacy class	"no" *vs.* "yes"	1.47	1.08-1.99	**0.01**	1.36	0.96-1.92	0.08
Caregivers heard about intestinal parasites	"no" *vs.* "yes"	1.36	0.98-1.88	**0.06**	0.98	0.67-1.43	0.92
Type of toilet in the household	Water pit latrine	1.00			1.00		
	Pit latrine	2.25	1.12-4.49	**0.07**	7.47	1.91-29.3	**0.006**
	No latrine	1.67	0.96-2.91		4.79	1.32-17.4	
Cleanliness of the toilet	"yes" *vs.* "no"	0.89	0.69-1.16	0.40			
Materials available in toilet	Sandals/slippers	0.86	0.45-1.65	0.66			
	Drum with water	1.36	0.96-1.91	**0.08**	0.37	0.12-1.21	0.10
	Brush	1.14	0.84-1.54	0.41			
	None of these	0.72	0.47-1.09	**0.12**	0.25	0.07-0.86	**0.03**
Soap available at handwashing facility	"yes" *vs.* "no"	0.87	0.55-1.37	0.55			
Trash outside the house	"yes" *vs.* "no"	0.72	0.54-0.97	**0.03**	0.80	0.55-1.16	0.24
Trash spread inside the house	"yes" *vs.* "no"	1.09	0.82-1.45	0.57			
Entirety of food covered	"yes" *vs.* "no"	1.06	0.78-1.43	0.72			
Flies in the kitchen	"yes" *vs.* "no"	1.05	0.76-1.44	0.78			
Caregiver's hands clean	"yes" *vs.* "no"	0.75	0.54-1.04	**0.09**	0.61	0.42-0.89	**0.01**
Caregiver is wearing shoes	"yes" *vs.* "no"	1.21	0.90-1.62	0.21			
Child's hand clean	"yes" *vs.* "no"	0.88	0.67-1.15	0.33			
Piles of dirty clothes in the house	"yes" *vs.* "no"	0.71	0.53-0.95	**0.02**	0.78	0.54-1.13	0.19
*E. coli* at point of use drinking water	"yes" *vs.* "no"	1.34	0.71-2.47	0.37			
E. coli at point of collection drinking water	"yes" *vs.* "no"	1.05	0.55-2.00	0.88			
Total coliforms at POC^d^ drinking water	"yes" *vs.* "no"	1.33	0.22-8.24	0.76			
Total coliforms at POU^e^ drinking water	"yes" *vs.* "no"	1.63	0.53-4.99	0.39			
Presence of undernutrition	"yes" *vs.* "no"	1.02	0.77-1.34	0.92			
Presence of nutritional deficiencies	"yes" *vs.* "no"	1.14	0.85-1.51	0.38			
Presence of diarrhoea	"yes" *vs.* "no"	1.16	0.81-1.65	0.41			

^a^Odds ratios were obtained from univariate mixed logistic regression models with random area intercepts, and *P*-values were obtained from Wald- and likelihood ratio tests. *P*-values <0.2 in the univariate analyses are marked in bold

^b^Adjusted odds ratios were obtained from a multivariable mixed logistic regression model with random area intercepts including all variables with *P*-values <0.2 in the univariate models along with gender and age group of the child and socio-economic category of the household. *P*-values were obtained from Wald- and likelihood ratio tests and values <0.05 are marked in bold

^c^Water, sanitation and hygiene

^d^Point of collection

^e^Point of use

Multivariate analysis showed that children in households with a simple pit latrine for defecation had seven times higher odds of being infected with intestinal parasites than those in households with a pour flush pit latrine (AOR = 7.47; 95% CI:1.91–29.3; *P* = 0.006). Children with caregivers having clean hands had significantly better odds of protection from intestinal parasitic infection (AOR = 0.61; 95% CI: 0.42–0.89; *P* = 0.01) than those with caregivers having dirty hands (Table 5).

### Water handling, water quality, sanitation, hygiene, and WASH promotion

Table D in the supplementary materials and Table 6 describes water handling, water quality, sanitation, hygiene practices and WASH promotion in the four study sites. Some 75.5% of the respondents depend on a communal village tap for drinking purposes and 20.7% had access to piped water in the house or yard. More than half (54.4%) of the respondents were confident about the safety of their available drinking water. 16.5% of the households reported treating their water at the point of use and one third (33.7%) reported disliking the taste of treated water.

**Table 6:** Hygiene behaviour and hygiene conditions [*N* = 1427]

Variables	Total [n (%)]	Surkhet A [n (%)]	Surkhet B [n (%)]	Dailekh [n (%)]	Accham [n (%)]	*P*-value*
Selected KAP^a^ indicators on hygiene						
Handwashing times						
<5 times	408 (28.6)	102 (29.3)	64 (17.5)	101 (28.4)	141 (39.4)	**<0.001**
5-10 times	935 (65.5)	225 (64.7)	251 (68.8)	251 (70.5)	208 (58.1)	
10-15 times	61 (4.3)	18 (5.2)	31 (8.5)	4 (1.1)	8 (2.2)	
> 15 times	23 (1.6)	3 (0.9)	19 (5.2)	0 (0.0)	1 (0.3)	
Handwashing with soap						
<5 times	1084 (76.0)	267 (76.7)	244 (66.8)	278 (78.1)	295 (82.4)	**<0.001**
5-10 times	337 (23.6)	79 (22.7)	118 (32.3)	78 (21.9)	62 (17.3)	
10-15 times	5 (0.3)	2 (0.6)	2 (0.5)	0 (0.0)	1 (0.3)	
> 15 times	1 (0.1)	0 (0.0)	1 (0.3)	0 (0.0)	0 (0.0)	
Handwashing^b^						
When they look dirty	862 (60.4)	224 (64.4)	254 (70.0)	172 (48.3)	212 (59.2)	**<0.001**
After going toilet	1402 (98.2)	342 (98.3)	356 (97.5)	354 (99.4)	350 (97.8)	0.21
After cleaning baby’s bottom	834 (58.4)	170 (48.8)	225 (61.6)	236 (66.3)	203 (56.7)	**<0.001**
Before eating	1039 (72.8)	259 (74.4)	298 (81.6)	295 (82.9)	187 (52.2)	**<0.001**
Before cooking	572 (40.1)	170 (48.8)	146 (40.0)	142 (39.9)	114 (31.8)	**<0.001**
There are not special occasions	2 (0.14)	0 (0.0)	1 (0.3)	0 (0.0)	1 (0.3)	0.58
Do not know	3 (0.21)	1 (0.3)	0 (0.0)	2 (0.6)	0 (0.0)	0.29
Types of latrines used						
Water pit latrine	1200 (84.1)	322 (92.5)	252 (69.0)	315 (88.5)	311 (86.9)	**<0.001**
Simple pit latrine	137 (9.6)	12 (3.5)	93 (25.5)	22 (6.2)	10 (2.8)	
No latrine	90 (6.3)	14 (4.0)	20 (5.5)	19 (5.3)	37 (10.3)	
Animal^c^ kept inside the household overnight	851 (59.7)	162 (46.5)	174 (47.8)	190 (53.4)	325 (90.8)	**<0.001**
Hygiene condition of latrine (observation)^d^						
Lower category	695 (48.7)	213 (61.2)	200 (54.8)	166 (46.6)	116 (32.4)	**<0.001**
Intermediate category	390 (27.3)	86 (24.7)	54 (14.8)	130 (36.5)	120 (33.5)	
High category	342 (24.0)	49 (14.1)	111 (30.4)	60 (16.9)	122 (34.1)	
Hygiene condition of handwashing facilities (observation)^e^						
Lower category	289 (50.5)	87 (39.9)	148 (67.0)	43 (43.9)	11 (31.4)	**<0.001**
Intermediate category	97 (17.0)	45 (20.6)	25 (11.3)	22 (22.4)	5 (14.3)	
High category	186 (32.5)	86 (39.5)	48 (21.7)	33 (33.7)	19 (54.3)	
Hygiene condition of household environment (observation)^f^						
Lower category	289 (50.5)	87 (39.9)	148 (67.0)	43 (43.9)	11 (31.4)	**<0.001**
Intermediate category	97 (17.0)	45 (20.6)	25 (11.3)	22 (22.4)	5 (14.3)	
High category	186 (39.5)	86 (39.5)	48 (21.7)	33 (33.7)	19 (54.3)	
Hygiene condition of kitchen (observation)^g^						
Lower category	481 (33.7)	187 (53.7)	174 (47.7)	75 (21.1)	45 (12.6)	**<0.001**
Intermediate category	637 (44.6)	128 (36.8)	120 (32.9)	206 (57.9)	183 (51.1)	
High category	309 (21.6)	33 (9.5)	71 (19.4)	75 (21.1)	130 (36.3)	
Personal hygiene of participating child and their caregivers (observation)^h^						
Lower category	611 (42.8)	153 (44.0)	210 (57.5)	167 (46.9)	81 (22.6)	**<0.001**
Intermediate category	424 (29.7)	92 (26.4)	72 (19.7)	130 (36.5)	130 (36.3)	
High category	392 (27.5)	103 (29.6)	83 (22.7)	59 (16.6)	147 (41.1)	
Information received on WASH^i^ promotion						
Received any information on water treatment and hygiene^j^	155 (10.9)	105 (30.2)	13 (3.6)	19 (5.3)	18 (5.0)	**<0.001**
Information on water treatment and hygiene changed behaviour	139 (89.7)	95 (90.5)	10 (76.9)	17 (89.5)	17 (94.4)	0.42
Behaviours that changed after receiving information on water treatment and hygiene^k^						
"I purchased a product for water treatment"	16 (1.1)	5 (1.4)	0 (0.0)	9 (2.5)	2 (0.6)	**<0.001**
"I am now regularly treating water"	32 (2.2)	20 (5.8)	2 (0.5)	9 (2.5)	1 (0.3)	**<0.001**
"I am now sometimes treating water"	27 (1.9)	23 (6.6)	1 (0.3)	2 (0.6)	1 (0.3)	**<0.001**
"I installed a handwashing station"	35 (2.4)	24 (6.9)	2 (0.8)	3 (0.8)	3 (0.8)	**<0.001**
"I do wash my hands more often"	36 (2.5)	23 (6.6)	3 (0.8)	4 (1.1)	6 (1.7)	**<0.001**
"I use soap to wash my hands more often"	90 (6.3)	63 (18.1)	6 (1.6)	15 (4.2)	6 (1.7)	**<0.001**
"I wash my hands at the critical times"	65 (4.6)	51 (14.7)	6 (1.6)	6 (1.7)	2 (0.6)	**<0.001**
"I regularly disinfect the water storage container with chlorine"	17 (1.2)	15 (4.3)	0 (0.0)	1 (0.3)	1 (0.3)	**<0.001**
"I regularly wash the water storage container with soap"	21 (1.5)	18 (5.2)	0 (0.0)	3 (0.8)	0 (0.0)	**<0.001**
"Other behaviour changed"	2 (0.1)	1 (0.3)	0 (0.0)	1 (0.3)	0 (0.0)	0.56
Household attended hygiene literacy class^l^	362 (25.4)	98 (28.2)	87 (23.8)	75 (21.1)	102 (28.5)	0.07
FCHVs^m^ or other health workers visited household	537 (37.6)	131 (37.6)	115 (31.5)	123 (34.5)	168 (46.9)	**<0.001**
Total times FCHVs/other health workers visited the household						
Once	468 (87.1)	102 (77.9)	99 (86.1)	112 (91.1)	155 (92.3)	**<0.001**
Twice	60 (11.2)	28 (21.4)	8 (7.0)	11 (8.9)	13 (7.7)	
Trice	3 (0.6)	0 (0.0)	3 (2.6)	0 (0.0)	0 (0.0)	
Four times	1 (1.2)	0 (0.0)	1 (0.9)	0 (0.0)	0 (0.0)	
Don`t remember	5 (0.9)	1 (0.8)	4 (3.5)	0 (0.0)	0 (0.0)	
Effectiveness: HLC^n^ or door to door visit						
Hygiene literacy class (HLC)	73 (23.0)	19 (25.0)	2 (2.5)	8 (11.9)	44 (45.8)	**<0.001**
Door to door visit	166 (52.2)	48 (63.2)	45 (57.0)	53 (79.1)	20 (20.8)	
Both	79 (24.8)	9 (11.8)	32 (40.5)	6 (9.0)	32 (33.3)	

^a^ Knowledge, attitude and practice

^b^ Multiple responses possible for the occasions of handwashing

^c^ Animals refers to any domestic animals such as chicken, goats, dogs, cats, cows, buffalos etc

^d^ A new variable for the observed hygiene condition of the toilet was created using factor analysis with conceptually similar categorical variables: (i) is the toilet clean; and (ii) are these materials available (sandals, drum with water, brush, none of these). The condition of toilet was then categorised into three categories with lower, intermediate and high hygiene category

^e^ A new variable for the observed hygiene condition of the handwashing facilities was created using factor analysis with four conceptually similar categorical variables: (i) are handwashing facilities in good condition; and (ii) are handwashing facilities clean (iii) is soap available (iv) is water available. The condition of handwashing facility was then categorised into three categories with lower, intermediate and high hygiene category

^f^ A new variable for the observed hygiene of the household environment condition was created using factor analysis with three conceptually similar categorical variables: (i) can you see trash spread outside the house; (ii) does the household have a garbage pit to dispose garbage (iii) can you see trash spread inside the house? The condition of household environment was then categorised into three categories with lower, middle and better hygiene

^g^ A new variable for the observed hygiene of the kitchen hygiene condition was created using factor analysis with four conceptually similar categorical variables: (i) are clean dishes kept high; (ii) is the entirety of food covered (iii) is there a rack to dry your utensils and dishes after washing and (iv) is there a significant number of flies in the kitchen (>10). The kitchen hygiene was then categorised into three categories with lower, intermediate and high hygiene category

^h^ A new variable for the observed personal hygiene of the caregiver and the participating child was created using factor analysis with four conceptually similar categorical variables of: (i) wearing shoes; (ii) hands are clean (iii) piles of dirty clothes lying around in the house. The personal hygiene was then categorised into three categories with lower, intermediate and high hygiene category

^i^ Water, sanitation and hygiene

^j^ Information from Helvetas (local INGO) or other organizations

^k^ Multiple responses possible

^l^ Hygiene literacy class conducted by female community health volunteers or other health workers

^m^ *FCHVs* Female community health volunteers

^n^ *HLC* Hygiene literacy class

We found that the majority of water samples from the point of collection and point of use were contaminated with *E. coli* (93.6 and 95.3%, respectively) and total coliform bacteria (99.4 and 98.8%, respectively). Five percent of water samples at the point of consumption met the WHO’s guidelines for microbial safety of drinking water (< 1 CFU *E. coli*/100 mL), 16.0% were in the low risk category (1–10 CFU *E. coli*/100 mL), 51.0% in the intermediate risk category (10–100 CFU *E. coli*/100 mL), and 28% in the high and very high risk categories (> 100 CFU *E. coli*/100 mL) [36].

We found that 6.3% of the households did not have latrines, and 93.7% had pit latrines. Almost half of the latrines (48.7%) were in a poor hygienic state. Three quarters (76.0%) of the respondents reported having washed their hands with soap less than five times per day prior to the day of the survey. The overall hygiene conditions were very low/ low in 64.0% of the surveyed households. Around 10% of the respondents reported having received information on water treatment and hygiene. Among those, 89.7% reported that the information changed their WASH behaviour, such as using soap more often for washing hands (Table 6).

### Prevalence of diarrhoea and associated risk factors

Table 7 presents the association of risk factors with diarrhoea. A total of 16.5% of children < 5 years suffered from diarrhoea within 7 days prior to the survey. The results from the multivariate regression analysis showed that children > 5 years old had significantly lower odds of diarrhoea (AOR = 0.39; 95% CI: 0.27–0.58; *P* = 0.01) than their younger counterparts. Children from the households experiencing a service interruption at the collection point of their main drinking water supply scheme of more than 1 week at the time of the visit had 2.87 higher odds of diarrhoea (AOR = 2.72; 95% CI: 1.18–6.31; *P* = 0.02) than children not experiencing such an interruption. Children of caregivers who were aware of the need for handwashing during critical times, such as when they looked dirty, were significantly better protected against diarrhoea (AOR = 0.47; 95% CI: 0.32–0.71; *P* = 0.01) than children of unaware caregivers. Children from households with clean latrines were significantly better protected against diarrhoea (AOR = 0.68; 95% CI: 0.47–0.98; *P* = 0.04) than those from other households. Similarly, children with visually clean hands were significantly better protected against diarrhoea than those with dirty hands (AOR = 0.62; 95% CI: 0.40–0.96; *P* = 0.03). Children living in households with a floor made of mud painted with animal dung had 2.29 times higher odds of suffering from diarrhoea than children living in households with a cement floor (AOR = 2.29; 95% CI: 1.20–4.37; *P* = 0.01).

**Table 7:** Association of risk factors with diarrhoea in univariate and multivariate logistic regression analysis

Risk factors [N (cases) = 492]		Univariate logistic regression^a^			Multivariate logistic regression^b^		
		OR	95% CI	*P-value*	aOR	95% CI	*P-value*
Age of child	< 5 years	1.00			1.00		
	> 5 years	0.47	0.34–0.66	**< 0.001**	0.39	0.27–0.58	**0.01**
Sex of child	Male	1.00			1.00		
	Female	1.08	0.82–1.44	0.58	1.14	0.82–1.58	0.43
Number of children in the household	< 5	1.00					
	> 5	2.18	0.66–0.18	0.20			
Caregivers can read/write	Can neither read or write	1.51	1.04–2.19		1.21	0.76–1.95	
	Can read only	0.22	0.03–1.62	**0.01**	0.22	0.03–1.79	0.15
	Can both read and write	1.00			1.00		
Involvement in the water supply system	“yes” vs. “no”	0.81	0.39–1.66	0.57			
In the community							
Socioeconomic status	Poor	1			1.		
	Middle	1.15	0.79–1.66	0.37	1.13	0.73–1.74	0.86
	Better	0.89	0.57–1.38		1.10	0.65–1.86	
Main drinking water source	Piped water in the house	1.00					
	Piped water in the village	0.81	0.50–1.31				
	Open source	1.07	0.33–3.49				
	Protected source	0.91	0.24–3.45	0.91			
	Unmanaged piped system	1.57	0.15–16.17				
	River, stream or canal	1.60	0.17–15.38				
Time to fetch drinking water	< 5 min	1.00			1.00		
	5–15 min	10.45	2.19–50.05		0.77	0.34–1.73	
	16–60 min	4.24	1.01–18.05	**< 0.001**	0.60	0.25–1.43	0.25
	> 60 min	3.13	0.73–13.44		0.26	0.05–1.44	
Interruption of the main drinking water source for more than a week	“yes” vs. “no”	2.92	1.49–5.71	**< 0.001**	2.72	1.18–6.31	**0.02**
Knowledge on factors that make water unsafe for drinking	Open unprotected source	2.39	1.71–3.33	**< 0.001**	0.66	0.37–1.17	0.16
	Open defecation	1.23	0.91–1.66	**0.17**	1.36	0.82–2.26	0.23
	Deforestation	1.05	0.48–2.29	0.90			
Method of drinking water treatment used	Boiling	0.94	0.45–1.97	0.87			
	Filtration with cloth	0.98	0.37–2.61	0.97			
	Use of filter (“yes” vs. “no”)	0.63	0.36–1.15	**0.12**	0.81	0.41–1.60	0.54
Handwashing with soap	< 5 times	2.49	0.45–13.89		5.52	0.49–61.63	
	5–10 times	0.56	0.39–0.82	**0.004**	1.35	0.77–2.38	0.31
	> 10 times	1.00			1.00		
Times of handwashing	When they look dirty (yes vs. no)	0.41	0.30–0.54	**< 0.001**	0.47	0.32–0.71	**0.01**
	After going to toilet (yes vs. no)	0.23	0.10–0.52	**< 0.001**	0.37	0.13–1.02	0.06
	After cleaning baby’s bottom (yes vs. no)	0.64	0.48–0.84	**< 0.001**	0.80	0.53–1.19	0.27
	Before eating (yes vs. no)	0.57	0.42–0.78	**< 0.001**	0.78	0.51–1.17	0.23
	Before cooking (yes vs. no)	0.91	0.68–1.22	0.55			
Animals inside home overnight	“yes” vs. “no”	1.06	0.78–1.44	0.72			
Information received on WASH	“no” vs. “yes”	0.71	0.40–1.24	0.23			
Handwashing station installed	“no” vs. “yes”	0.57	0.17–1.89	0.35			
Wash hands during critical times	“yes” vs. “no”	0.91	0.41–2.01	0.83			
Attended hygiene literacy class	“no” vs. “yes”	0.47	0.32–0.68	**< 0.001**	0.76	0.46–1.25	0.29
Caregivers heard about intestinal parasites	“no” vs. “yes”	0.72	0.49–1.05	**0.09**	1.06	0.59–1.90	0.84
Awareness on measures against intestinal parasites	Wash hands with soap	0.99	0.62–1.58	0.95			
	Wear shoe	0.85	0.43–1.68	0.64			
	Drink clean water	0.81	0.53–1.22	0.30			
	Deworming	0.61	0.36–1.04	**0.07**	1.29	0.58–2.84	0.53
Type of toilet in the household	Water pit latrine	1.67	0.85–3.27		0.84	0.15–4.78	
	Pit latrine	0.82	0.47–1.42	**0.008**	0.69	0.13–3.79	0.75
	No latrine	1.00			1.00		
Cleanliness of the toilet	“yes” vs. “no”	0.57	0.43–0.77	**< 0.001**	0.68	0.47–0.98	**0.04**
Materials available in toilet	Sandals/slippers	0.48	0.17–1.37	**0.17**	1.18	0.37–3.80	0.78
	Drum with water	0.77	0.54–1.08	**0.13**	1.35	0.28–6.69	0.71
	Brush	0.46	0.31–0.68	**< 0.001**	0.92	0.56–1.52	0.75
	None of these	1.41	0.93–2.13	**0.10**	1.16	0.22–6.04	0.86
Trash outside the house	“no” vs. “yes”	0.62	0.44–0.86	**0.01**	1.01	0.61–1.68	0.98
Trash spread inside the house	“yes” vs. “no”	1.33	0.98–1.81	**0.07**	0.77	0.43–1.23	0.27
Entirety of food covered	“yes” vs. “no”	0.59	0.48–0.80	**< 0.001**	0.79	0.51–1.23	0.30
Flies in the kitchen	“yes” vs. “no”	0.97	0.67–1.40	0.85			
Caregiver’s hands clean	“yes” vs. “no”	0.53	0.38–0.73	**< 0.001**	0.84	0.51–1.38	0.50
Caregiver wearing shoe	“yes” vs. “no”	0.57	0.41–0.77	**< 0.001**	0.96	0.64–1.46	0.87
Child’s hand clean	“yes” vs. “no”	0.45	0.34–0.61	**< 0.001**	0.62	0.40–0.96	**0.03**
Piles of dirty clothes in the house	“yes” vs. “no”	1.34	0.98–1.85	**0.07**	0.71	0.43–1.18	0.19
*E. coli* at point of use drinking water	“yes” vs. “no”	3.59	1.10–11.69	**0.03**	2.19	0.62–7.66	0.22
Total coliform at POU drinking water	“yes” vs. “no”	1.34	0.16–11.34	0.79			
Presence of undernutrition	“yes” vs. “no”	1.12	0.83–1.51	0.47			
Presence of intestinal parasites	“yes” vs. “no”	1.19	0.84–1.70	0.33			
Floor materials	“mud” vs. “cement”	2.98	1.71–5.20	**0.01**	2.29	1.20–4.37	**0.01**

^a^ Odds ratios were obtained from univariate mixed logistic regression models with random area intercepts, and P-values were obtained from Wald- and likelihood ratio tests. *P*-values < 0.2 in the univariate analyses are marked in bold

^b^ Adjusted odds ratios were obtained from a multivariate mixed logistic regression model with random area intercepts including all variables with *P*-values < 0.2 in the univariate models along with gender and age group of the child and socio-economic category of the household. *P*-values were obtained from Wald- and likelihood ratio tests and values < 0.05 are marked in bold

### Child health and health-seeking behaviours

Table E in the supplementary materials shows the percentage distribution of child health records, health-seeking behaviours, and knowledge, attitude and practices related to health and hygiene. A total of 49.9% of the children from < 6 months to 5 years were reported to have been sick within 7 days prior to the survey. Respiratory illnesses and fevers were most common (both 40.4%), followed by diarrhoea (16.5%).

A majority (82.7%) of the respondents knew that contaminated water can cause diarrhoea. However, a majority (78.9%) had never heard about intestinal parasites. The proportions of caregivers who were aware that handwashing with soap might prevent intestinal parasitic infections were 11.1%, wearing shoes 5.0%, drinking clean water 16.6%, and undergoing regular deworming treatment 11.1%.

## Discussion

Our findings highlight alarming health conditions among children in the remote areas of rural Nepal where the study took place. While more than half of the surveyed children were infected with parasites and suffered from undernutrition and nutritional deficiencies, the prevalence of diarrhoea was slightly lower. Our analysis identified specific risk factors for each of these health outcomes.

### Undernutrition

The high prevalence of undernutrition in our study sites could be explained by high poverty rates. Undernutrition was linked less to hygiene-related risk factors and more to the low socioeconomic status of the household and poor nutrition. These findings are in line with the results of recent randomised evaluations of WASH and nutrition interventions, which found that nutritional interventions significantly reduced child stunting or thinness, whereas WASH interventions, delivered either separately or in a combined fashion, showed no such effects on child health outcomes [37–39]. Although the relationship between undernutrition and intestinal parasitic infections is not well understood, undernutrition may be caused by recurring infections in the gut, which limit the proper absorption of calories and nutrients [40, 41]. Our findings, which identified an association between undernutrition and intestinal parasitic infection, are in agreement with studies conducted elsewhere [41, 42]. However, in contrast to a previous study conducted in Bangladesh, our study did not identify diarrhoea infection as a risk factor for undernutrition [43]. Because this study relies on a cross-sectional design, we do not have any longitudinal information on the frequency and severity of diarrhoea cases occurring in the study population. We expect that chronic diarrhoea and environmental enteropathy are likely linked with undernutrition; however, this hypothesis cannot be confirmed in the present study [39, 44, 45].

We observed that unsafe water was used to wash feeding and storage containers, unhygienic kitchen cloths were used to dry children’s utensils, caregivers did not wash their hands with soap while preparing and feeding children and food was not hygienically stored. In addition, 76.8% of the households had flies indoors and in their surroundings. The recurrent food-borne infections are likely to have contributed to nutritional deficiency, environmental enteropathy, and consequent undernutrition [39, 41, 46, 47]. Similar observations of unsafe WASH practices and inadequate food hygiene were reported in a study conducted elsewhere in Nepal [48].

### Clinical signs of nutritional deficiencies

The prevalence of 63.9% of children having at least one clinical sign for a nutritional deficiency was high. Due to the dearth of studies conducted on children with clinical signs of nutritional deficiencies in Nepal and other similar contexts, it is difficult draw meaningful comparisons with other studies. The most frequently encountered sign of a nutritional deficiency, pale conjunctiva indicates iron-deficiency and anaemia and, thus, can be related to the lack of animal protein in the diet.

In contrast to our findings on the risk factors associated with undernutrition, clinical signs of nutritional deficiencies were significantly associated with water quality and various hygiene factors. Our analysis identified a significant protective association with handwashing, improved latrine cleanliness, better hygiene in the kitchen, and household’s own production of food. A higher risk for a nutritional deficiency was associated with poor water quality at the source, keeping animals inside the house overnight, and the low personal hygiene of caregivers and of children. Further in-depth research is required to provide more insight into these issues.

### Intestinal parasitic infections

The high prevalence of intestinal parasitic infections among children in our study is similar to or higher than the rate reported in studies conducted in other areas of Nepal [16, 18, 49]. The higher infection rates may be explained by the fact that our study areas were located in extremely remote and hilly areas with difficult road access and a lack of infrastructure, which together results in a low level of access to basic health and WASH services [16, 18, 49]. Our analysis showed that children from households with simple pit latrines had higher odds of developing an intestinal parasitic infection than did those with water sealed latrines. The effect of inadequate sanitary conditions on intestinal parasitic infections was also documented in a systematic review and meta-analysis conducted by Ziegelbauer et al. (2012) [50].

The cleanliness of caregivers’ hands was identified as a significant risk factor for children’s parasitic infections, suggesting that caregivers’ hands play a critical role in transferring parasites from the household environment to their children. We observed poor handwashing conditions and a limited presence of soap and water at the handwashing stations. The importance of clean hands to preventing parasitic infections is in agreement with previous studies conducted in eastern Nepal [16, 51]. There is strong evidence that a high load of pathogens in the household environment and inadequate handwashing increase the density of pathogens on caregivers’ hands [52]. The association between inadequate sanitation, insufficient hygiene and infections with intestinal parasites has also been documented by studies conducted in other parts of Nepal [16, 49, 53].

### Diarrhoea

We observed a very strong association between diarrhoea prevalence and the children living in a house with a mud floor, similar to studies conducted in Bangladesh [54, 55]. The cultural practice of painting mud floors in the home with animal dung remains widespread in the study area, indicating a need for this potential driver of exposure to be given increased attention. We hypothesize that the practice of painting floors with cow dung leads to a high load of diarrhoea causing pathogens in the household environment at orders of magnitude higher than concentrations found in drinking water, thus masking the impact of clean drinking water on children’s health. Children playing on the floor inside or around their houses are at high risk of ingesting pathogens [52, 56]. This assumption is confirmed by several studies that report an association between *E. coli* contamination of the floor with the disposal of faeces and the presence of animals close to the household. Kwong et al. reported that 35% of children put their hands in their mouths after touching soil particles, putting them at risk of contamination [55]. Additionally, we observed that animals were often kept in or near the home and brought indoors overnight. Such practices have been shown to increase exposure to faecal contamination in the household environment in other rural settings [15, 57–59]. Other studies conducted in India and Bangladesh highlighted the importance of faecal contamination of animal origin in the domestic environment, including source and stored drinking water, hands, and soil [43, 44].

A strong association was also found between diarrhoea incidence and reported interruptions of the water supply. Underlying reasons for this might be the subsequent lack of water for hygienic purposes. In addition, intermittent water services present an increased need for storage at the household level and, therefore, the potential for recontamination. The risk of pathogen infiltration into the piped network, might also be greater during such low-pressure events [58, 59]. Similar results were reported in a study conducted in low- and middle-income countries, which reported that the provision of high-quality piped water, sewer connections, and the use of water filters were associated with considerable reductions in diarrhoea [60].

## Conclusion

In our study, more than half of the children living in the remote hilly areas of Nepal suffered from impaired nutritional status, nutritional deficiencies, intestinal parasitic infections, and to a lesser degree, diarrhoea disease. A better nutritional status of children was only indirectly linked to WASH factors. The odds of children having parasitic infections and diarrhoea incidence were both highly associated with poor hand hygiene and inadequate sanitation. Keeping animals in the household overnight and painting mud floors with animal dung were identified as important risk factors for child diarrhoea and nutritional deficiencies. Consequently, interventions to reduce the load of pathogens transmitted by animals into the household environment could be promising for improving children’s health and require further investigation. To reduce diarrhoea risk, our findings also highlight the importance of having access to a safe, reliable and continuous supply of water, which is necessary for adequate hygiene practices.

## Supplementary information

**Additional file 1.** Questionnaire for the caretakers.

**Additional file 2: Table A**. characteristics of the study population.

**Additional file 3: Table B**. Detailed information on Children’s nutritional status in the four study sites.

**Additional file 4: Table C**. Nutrition provided to children between 6 months and 10 years.

**Additional file 5: Table D**. Water supply, water handling and water quality.

**Additional file 6: Table E**. Child health, health seeking behaviour and awareness on health protecting behaviours.

## Data Availability

The dataset and the questionnaire supporting the conclusions are available from the corresponding author on reasonable request.

## Abbreviations

AOR: Adjusted odds ratio
BMIZ: Body Mass Index Z score
EPG: Eggs per gram
FAO: Food and Agricultural Organisations
HAZ: Height for age Z score
KAP: Knowledge, attitude and practices
SDC: Sustainable development goals
WAZ: Weight for age Z score
WASH: Water, sanitation, and hygiene
WARM-P: Water resource management programme
WHO: World Health Organisation
