# Supplementary material for: Association of nutrition, water, sanitation and hygiene practices with children’s nutritional status, intestinal parasitic infections and diarrhoea in rural Nepal: a cross-sectional study
Source: BMC Public Health. 2020 Aug 15;20:1241. doi: 10.1186/s12889-020-09302-3 (PMC7429949; doi:10.1186/s12889-020-09302-3)
Supplement: Supplementary file 1 — Additional file 1. Questionnaire for the caretakers. [file 12889_2020_9302_MOESM1_ESM.pdf]

Questionnaire Main Caretaker

introduction

All the information you provide is confidential and your name will not be disclosed anywhere. The results will be treated anonymously. Participation in this study is voluntary. You don't have to take part if you don't want to. You don't have to answer any question you don't want to, and you can stop the interview at any time. If you decide not to participate there will not be any negative consequences.

Do you have any questions? Do you agree to participate in this study?
If you have any further questions you can contact Madan Bhatta from Helvetas. The phone number of the Helvetas office in Surkhet is: 083521092 / 083521093

Administer informed consent. If subject agrees to participate, proceed to questionnaire

◯ Yes

◯ No

beginning

Request the household to provide you a sample of drinking water. Pour the water directly into the whirlpack as if a glass of drinking water would be filled.

The sample should be taken in the same way the household would prepare a glass of drinking water (for example from drinking water storage, filter reservoir, thermos etc.)

Now you can begin the interview!

◯ Proceed

Name of interviewer

◯ Mina Sunar

◯ Ramesh Yogi

◯ Ammar Bahadur Nepali

◯ Prakash Bhandari

◯ Lalita Bhadari

◯ Surendra Mahato

◯ Durga Paudel

Enter the household ID (HHID)

Copy that Household ID in your notebook in case you need to take notes

____

Please select the district

◯ Surkhet 1

◯ Surkhet 2 (Control)

◯ Dailekh

◯ Achham

Select the municipality/VDC

____

Select the ward number

____

Select the name of the area

- ◯ area A
- ◯ area B
- ◯ area C
- ◯ area D

A - Household Information

A-Household information

Name of person interviewed

____

What is the gender of the repondent?

Please indicate without notifying the respondent

- ◯ Male
- ◯ Female

What is the age of the respondent?

Enter number / if unknown: approximate the age: ."60-70"

____

What is your mobile phone number?

Enter mobile phone number / Enter 999 if no mobile phone

____

How many people in TOTAL, including you, live in your household?

Including respondent

____

How many children are 0 to 10 years in this household?

Enter number

____

How many children are below the age of 5?
Enter number

____

How many children go to school?
Enter number

____

Are you able to read or write?

- ◯ Can neither read or write
- ◯ Can read only
- ◯ Can both read or write

What is the highest education level you have completed?
Do not read out - select one answer given by respondent

- ◯ Informal education
- ◯ Primary
- ◯ Secondary
- ◯ College and higher
- ◯ None/ Don't know

What is the occupation of the head of the household?
Do not read out - select one answer given by respondent

- ☐ Agriculture
- ☐ Small business
- ☐ Daily laborer
- ☐ Employed
- ☐ Government service
- ☐ Other independent work
- ☐ Retired with pension
- ☐ None

What is the occupation of the spouse of the household head?

Do not read out - select one answer given by respondent

- [ ] Agriculture
- [ ] Service
- [ ] Small business
- [ ] Daily laborer
- [ ] Other independent work
- [ ] Retired with pension
- [ ] None
- [ ] Foreign employment
- [ ] No spouse (=single or widow)

What is the ethnicity of this household?

Do not read out - select one answer given by respondent

- ◯ Dalit
- ◯ Janajati
- ◯ Bramihin, Chhetr, Thakuri
- ◯ Other

Specify other ethnicity

Is anyone in this household involved in the water supply system in this community ?

- ◯ Yes
- ◯ No

Does anyone in this household hold a leadership position in this community?

- ◯ Yes
- ◯ No

What would you say is the biggest concern for people living in this VILLAGE?

Do not read out - select one answer given by respondent

- ◯ Health and healthcare services Sanitation (toilets and drainage)
- ◯ Transportation and roads
- ◯ Security and crime
- ◯ Electricity services
- ◯ Jobs and unemployment
- ◯ Education
- ◯ Support for agriculture
- ◯ Water supply services
- ◯ Don't know/ could not respond
- ◯ Other major concern

Please specify other:

____

B - Wealth index

B - Wealth index

About how much does your household spend PER MONTH on regular expenses (regular expenses = food, transport, clothing, and school fees) ?

Insert number in NPR per month

____

Does anyone from your household own/ have any of these items?

Read out all options

- ☐ Electricity in the house
- ☐ Radio
- ☐ TV
- ☐ Solar panel
- ☐ Mobile phone
- ☐ Bicycle
- ☐ Motorbike
- ☐ Car
- ☐ Fridge
- ☐ Watch
- ☐ None of this

Does the household have an electricity connection?

- ◯ Yes
- ◯ No

What kind of fuel do you use mainly for cooking?

Do not read out - select all answers given by respondent

- ☐ Wood
- ☐ Charcoal
- ☐ Kerosene
- ☐ Gas
- ☐ Electricity

Are you the owner of your house?

- ◯ Own house
- ◯ Rent house

How many rooms does your house have?

____

How much land does your family own?

Enter area owned by the household in Ropanis
If no area is owned enter "0"
If don't know enter "999"

____

C - Water handling and hygiene

C-Water handling and hygiene

Which water source do you currently use as MAIN drinking water source?

Do not read out

- ◯ Piped water in the house or yard
- ◯ Piped water in the village
- ◯ Rainwater harvesting
- ◯ Open source (dug well, pond, spring)
- ◯ Protected source (well, spring)
- ◯ Unmanaged piped system
- ◯ River, Stream or Canal
- ◯ Lake
- ◯ Bottled Water

Do you currently also use other water sources for drinking?

- ◯ Yes
- ◯ No

Which other water sources for drinking water do you currently use?
Do not read out - select all answers given by respondent

- ☐ Piped water in the house or yard
- ☐ Piped water in the village
- ☐ Rainwater harvesting
- ☐ Open source (dug well, pond, spring)
- ☐ Protected source (well, spring)
- ☐ Unmanaged piped system
- ☐ River, Stream or Canal
- ☐ Lake
- ☐ Bottled Water

How often does WUSC meet with water users to discuss issues about the water system?

- ◯ Monthly
- ◯ Bi-monthly
- ◯ Once every 3 months
- ◯ Once every 6 months
- ◯ Once per year
- ◯ As needed
- ◯ Never
- ◯ Don't know/ no answer

How long does a trip (back and forth) to your main drinking water source take, including time required to queue and to fill the containers?
Insert time in Minutes

____

Is your main drinking water source functioning now?

- ◯ Yes, functioning well
- ◯ Yes, functioning but not well
- ◯ No, not functioning

If main drinking water source needed repairs, how confident are you that the problem could be fixed within 1 week?

- Very confident
- Somewhat confident
- Not confident at all
- Don't know/ no answer

Is there a village maintenance worker (VMW) to look after your main drinking water source?

- Yes
- No

Can you get help from the VMW to repair main drinking water source when you need it?

- Yes
- Maybe
- No
- Don't know/no answer

In the last 6 months, were there any times when water from main drinking water source was not available for more than one week?

- Yes
- No

How many days did the interruption last?

____

Do you think the main drinking water source will be functional one year from now?

- Yes
- No

Instructions: Think about the home, animals or furniture that you own or co-own with someone, and the experiences and feelings associated with the statement 'THIS IS MY (OUR) HOUSE!' The next 10 questions deal with the 'sense of ownership' that you feel for the water system in your village. How true are the following statements for you? (to be adapted after pretest)

How much do you agree with the following statement? This is MY water system.

Use five dots scale

- Not at all true
- Hardly true
- Rather true
- Mostly true
- Very true

How much do you agree with the following statement? This is OUR COMMUNITY'S water system.

Use five dots scale

- ◯ Not at all true
- ◯ Hardly true
- ◯ Rather true
- ◯ Mostly true
- ◯ Very true

How much do you agree with the following statement? It is hard for me to think about this water system as MINE.

Use five dots scale

- ◯ Not at all true
- ◯ Hardly true
- ◯ Rather true
- ◯ Mostly true
- ◯ Very true

How do you perceive the taste of drinking water from your main drinking water source right now?

Do not read out

- ◯ Very good
- ◯ Good
- ◯ Medium
- ◯ Bad
- ◯ Very bad

How safe do you think your main drinking water source is for drinking?

Do not read out

- ◯ Very safe
- ◯ Quite safe
- ◯ Neither safe nor risky
- ◯ A bit risky
- ◯ Very risky

What do you think could make your water unsafe for drinking?

Do not read out - select answer that match with categories

- [ ] Open unprotected source
- [ ] Unmanaged system/fittings chamber pipe
- [ ] Open defecation
- [ ] Settlement above source
- [ ] Deforestation
- [ ] Don't know
- [ ] Other...

Please specify other:

Which methods for water treatment do you know?

Do not read out

- [ ] Boiling
- [ ] Filtration with a cloth
- [ ] Flocculation and sedimentation
- [ ] Chlorination
- [ ] Sodis
- [ ] Use of Filter
- [ ] Other (specify)
- [ ] Do not know any

Please specify other:

Can you explain to me the procedures of the different methods (the ones the interviewee knows) for water treatment?

Let the person explain the different methods for water treatment

- ○ Good explaination of at least 4 methods
- ○ Good explaination of 3 methods
- ○ Good explaination of 2 methods
- ○ satisfactory explanation of 1 method
- ○ Cannot explain well
- ○ Don't know any

Do you use any method to treat your drinking water?

- ◯ Yes
- ◯ No

Which methods for water treatment did you use for drinking water treatment in the last 2 weeks?

Do not read out

- ☐ Boiling
- ☐ Filtration with a cloth
- ☐ Flocculation and sedimentation
- ☐ Chlorination
- ☐ Sodis
- ☐ Use of Filter
- ☐ Other

Specify other treatment

____

Who in your family is mainly responsible for water treatment?

- ☐ Wife
- ☐ Husband
- ☐ Daughter
- ☐ Son
- ☐ Other

How often did you treat your drinking water in the past 2 weeks?

- ☐ Every day
- ☐ Sometimes
- ☐ Only during rainy season
- ☐ Only for sick poeple
- ☐ Only for babies and children below 5 years
- ☐ Never
- ☐ Do not know

How much do you like or dislike drinking treated water?

Use 5 dots scale

- ◯ I dislike it very much
- ◯ I rather dislike it
- ◯ Average
- ◯ I rather like it
- ◯ I like it very much

Do you think that treating your drinking water is worthwhile?

Use 5 dots scale

- ◯ Never worthwile
- ◯ Rarely worthwile
- ◯ Sometimes worthwile
- ◯ Mostly worthwile
- ◯ Always worthwile

How much would people who are important to you approve or disapprove if you treated your drinking water?

Use 5 dots scale

- ◯ They would disapprove very much
- ◯ They would rather disapprove
- ◯ Neither approve nor disapprove
- ◯ They would rather approve
- ◯ They would approve very much

How many people who are important to you (your family, friends etc.) treat their water before drinking?

Use 5 dots scale / do not read out the %, it is just a help for you to visualise the number of people

- ◯ (Almost) nobody (0%)
- ◯ Some of them (25%)
- ◯ Half of them (50%)
- ◯ Most of them (75%)
- ◯ (Almost) all (100%)

How sure are you that you can always treat your water before drinking, even if this may be difficult sometimes?

Use 5 dots scale

- ◯ Not at all sure
- ◯ Hardly sure
- ◯ Rather sure
- ◯ Mostly sure
- ◯ Very sure

Have you made a detailed plan how to overcome difficulties to water treatment (e.g. when you run out of chlorine, you have no time etc.)?

Use 5 dots scale

- ◯ No detailed plan at all
- ◯ No detailed plan
- ◯ Quite detailed plan
- ◯ Detailed plan
- ◯ Very detailed plan

In the last four weeks, how often did it happen that you wanted to drink water, but you forgot to treat it in time?

Use 5 dots scale

- ◯ (Almost) never forget
- ◯ Sometimes forget
- ◯ About half of the times forget
- ◯ Almost always forget
- ◯ Never treats drinking water (always forget)

How important is it for you to treat your water before drinking

Use 5 dots scale

- ◯ Not at all imporant
- ◯ Little important
- ◯ Rather important
- ◯ Important
- ◯ Very important

How strongly do you intend to always treat your water before drinking?

Use 5 dots scale

- ◯ Not at all
- ◯ Little
- ◯ Rather
- ◯ Strongly
- ◯ Very strongly

How true is the following statement for you? Treating my drinking water is something... I do automatically.

Use 5 dots scale

- ◯ Not at all true
- ◯ Hardly true
- ◯ Rather true
- ◯ Mostly true
- ◯ Very true

Collection and Transport containers

What kind of containers do you use to collect & transport water from the source?

- ☐ Gagri silver
- ☐ Gagri brass
- ☐ Gagri copper
- ☐ Gagri plastic
- ☐ Jerrycan plastic
- ☐ Plastic bucket with large opening
- ☐ Alu bucket with large opening
- ☐ Claypot
- ☐ Other

Please specify other:

Do you clean your container for transport?

- ◯ Yes
- ◯ No

How often do you clean the container used for transport of water?

- ◯ Every day
- ◯ Every second day
- ◯ At least once per week
- ◯ Less often than once per week

How do you clean the container used for transport of water?

- ◯ I use water or water and sand
- ◯ I use Chlorine to disinfect it almost always
- ◯ I use Chlorine to disinfect it sometimes
- ◯ I wash it almost always with soap or ash
- ◯ I wash it sometimes with soap or ash

Do you use the same container for water transport and water storage?

- ◯ Yes
- ◯ No

Storage containers

What kind of containers do you use to store the drinking water ?

- [ ] Ceramic Water Filter
- [ ] Small Container with tap
- [ ] Small Container without tap
- [ ] Large Container with tap
- [ ] Large Container without tap
- [ ] Other

Please specify other:

____

Do you clean your container for storage?

- ◯ Yes
- ◯ No

How often do you clean your safe storage container?

- ◯ Every day
- ◯ Every second day
- ◯ At least once per week
- ◯ Less often than once per week

How do you clean your safe storage container?

- ◯ I use water or water and sand
- ◯ I use Chlorine to disinfect it almost always
- ◯ I use Chlorine to disinfect it sometimes
- ◯ I wash it almost always with soap or ash
- ◯ I wash it sometimes with soap or ash

YESTERDAY, can you tell me how many times you washed your hands?

Enter the number of times the respondent washed hands yesterday.
If zero, enter "0"
If don't know, enter "999"

____

When you washed your hands yesterday, how often did you use soap or ash?

____

During which times did you wash your hands yesterday?

Do not read out, multiple answer possible

- ☐ When they look dirty
- ☐ After going to toilet
- ☐ After cleaning baby's bottom
- ☐ Before eating
- ☐ Before cooking
- ☐ There are no special occasions
- ☐ Never
- ☐ Do not know

Where do members of your family usually go for defaecation?

Do not read out, if answer unclear give options

- ◯ They use the bushes
- ◯ A shared simple pit latrine
- ◯ A shared water sealed toilet
- ◯ Household's own simple pit latrine
- ◯ Household's own water sealed toilet

Do you keep the animal safe inside your house over night ?

- ◯ Yes
- ◯ No

D - Information on WASH Promotion

D - Information on WASH Promotion

Have you received any information on water treatment and hygiene from Helvetas or others in the last 2 months?

- ◯ Yes
- ◯ No

Did the information on water, hygiene and sanitation change your behavior?

- ◯ Yes
- ◯ No

Which behaviour did you change after your received information on water treatment and hygiene?
Do not read out; listen to explanation and tick applicable boxes

- ☐ I purchased a product for water treatment
- ☐ I am now regularly treating water
- ☐ I am now sometimes treating water
- ☐ I installed a handwashing station
- ☐ I do wash my hands more often
- ☐ I use soap to wash my hands
- ☐ I wash my hands at the critical times
- ☐ I regularly disinfect the water storage container with chlorine
- ☐ I regularly wash the water storage container with soap
- ☐ Other behaviour changed

Please specify other:

____

Has any of your family member attended the Hygiene Literacy class conducted by FCHV or other health workers ?

- ◯ Yes
- ◯ No

Had FCHV pr other health workers visited your Household?

- ◯ Yes
- ◯ No

How many times in the last year has a FCHV or other health workers visited your household in total?

____

Which method between HLC and door to door visit did you find more effective?

- ◯ HLC
- ◯ Door to door visit
- ◯ Both
- ◯ None

E - Child Health

E - Child Health

What are the causes for diarrheal diseases?

Let the respondent explain the cause, tick the selection which matches the explanation best

- ☐ Some pathogens
- ☐ Faecal pathogens
- ☐ Dirty hands
- ☐ Dirty food
- ☐ Dirty water
- ☐ Explanation does not correspond with real cause

How high or low are the chances that you or your children get sick if you drink untreated water?

Use 5 dots scale

- ◯ Very low
- ◯ Rather low
- ◯ Average
- ◯ Rather high
- ◯ Very high

Imagine your child below 5 years has diarrhea, how severe would be the impact on his life and development?

Only read options if answer unclear. Use 5 dots scale

- ◯ Not at all severe
- ◯ Hardly severe
- ◯ Rather severe
- ◯ Severe
- ◯ Very severe

» Children's illnesses

Note the ID Code of child

____

How old is child?

Enter number of year

____

Enter number of months

____

What is the gender of child ?

- ◯ female
- ◯ male

Type of illnesses

Has your child suffered from any of the following illnesses in the past 7 days?

	Yes	No
**Fever**	○	○
**Cough**	○	○
**Respiratory difficulties**	○	○
**Diarrhoea (passage of liquid stool more than 3 times per day)**	○	○
**Blood in stool**	○	○
**Mucus in stool**	○	○
**Blood in urine**	○	○

Was the child sick within the past seven days?

○ Yes

○ No

Did you seek medical advice for the condition/illness of your child?

○ Yes

○ No

Where did you seek medical advice or treatment?

☐ hospital

☐ health center or health post

☐ community health worker

☐ pharmacy

☐ self-treatment, traditional medicine

☐ other

Why did you not seek medical advice?

☐ I do not have good access to a health facility

☐ I do not like services/personnel/structures at the health facility

☐ I do not have money to go to the health facility

☐ I prefer self-treatment or traditional medicine

☐ It was not necessary to go to the health facility

☐ I did not have permesssion to go to the health facility

☐ Other

Have you ever heard of 'intestinal parasites'?

- ◯ Yes
- ◯ No

What can be done against intestinal parasites

Do not promt

- ☐ Wash hands with soap
- ☐ Cut finger nails
- ☐ Wear pants, trousers
- ☐ Wash fruits and vegetables before consumption
- ☐ Wear shoes
- ☐ Drink clean water
- ☐ Take medication
- ☐ Other

F - Nutrition

F - Nutrition

Did you breastfeed your child?

- ◯ Yes
- ◯ No

How many months did you breastfeed your child?

____

Did you feed your child any other liquids or foods during the first six months in addition to mother breastfeeding?

- ◯ Yes
- ◯ No

After how many months of age did you start to provide additional weaning food to your child?

____

Usually how many meals do your children eat per day?

____

Do you give your child other things to eat, for example sweets or snacks in addition to the usual meals?

- ◯ Yes
- ◯ No

What do you give?

____

Do you give your child any food supplements, for example (sarbottam pitho) in addition to the usual meals?

- ◯ Yes
- ◯ No

What supplements do you give?

____

How many meals per day do the adults in your household eat?

____

» Type of food

In the past week, can you think about whether your family consumed any of these foods?

Starchy staple food (rice, grain, potato, etc.)

- ◯ three times per day
- ◯ twice per day
- ◯ once per day
- ◯ every second day
- ◯ two times per week
- ◯ once per week
- ◯ less than once per week
- ◯ Sometimes
- ◯ not at all

Beans, peas or lentils

- ◯ three times per day
- ◯ twice per day
- ◯ once per day
- ◯ every second day
- ◯ two times per week
- ◯ once per week
- ◯ less than once per week
- ◯ Sometimes
- ◯ not at all

Nuts or seeds

- three times per day
- twice per day
- once per day
- every second day
- two times per week
- once per week
- less than once per week
- Sometimes
- not at all

Dairy products (for example milk, yoghurt)

- three times per day
- twice per day
- once per day
- every second day
- two times per week
- once per week
- less than once per week
- Sometimes
- not at all

Meat or fish

- three times per day
- twice per day
- once per day
- every second day
- two times per week
- once per week
- less than once per week
- Sometimes
- not at all

Eggs

- three times per day
- twice per day
- once per day
- every second day
- two times per week
- once per week
- less than once per week
- Sometimes
- not at all

Leafy green vegetables (sauce included)

- three times per day
- twice per day
- once per day
- every second day
- two times per week
- once per week
- less than once per week
- Sometimes
- not at all

Other vegetables

- three times per day
- twice per day
- once per day
- every second day
- two times per week
- once per week
- less than once per week
- Sometimes
- not at all

Fruits

- ◯ three times per day
- ◯ twice per day
- ◯ once per day
- ◯ every second day
- ◯ two times per week
- ◯ once per week
- ◯ less than once per week
- ◯ Sometimes
- ◯ not at all

Do you produce your own food?

- ◯ Yes
- ◯ No

Is your harvest enough for meeting your household's food needs all year long?

- ◯ Yes
- ◯ No

For how many months do you have to buy extra food (not sufficient production from own agricultural activities)?

- ◯ Up to 3 months
- ◯ 3 to 6 months
- ◯ More than 6 months
- ◯ Do not need to buy any food

G - Observation through the interviewer (your own observation)

G - Observation through the interviewer (your own observation)

What type of walls does the main house have?

- ◯ Stone with mud
- ◯ Stone with cement
- ◯ Brick with cement
- ◯ Wood planks
- ◯ Corrugated iron
- ◯ Cement

What type of roof does the main house have?

- ◯ Straw
- ◯ Roof tiles/ Stone slates
- ◯ CGI Sheet
- ◯ RCC
- ◯ Made from mud

What type of floor does the main house have?

- ◯ Earth
- ◯ Cement
- ◯ Floor tiles

Can you show me the product you use for water treatment?

- ☐ Black kettle (for boiling)
- ☐ Chlorine bottle available
- ☐ Water filter available
- ☐ SODIS bottle available
- ☐ PUR (flocc & sedimentation) available
- ☐ Cloth for filtration available
- ☐ No product for water treatment present

» Condition of water transport container

In which condition is the container used for water transport?

Is the water transport container clean?

- ◯ Yes
- ◯ No

Does the water transport container have a lid?

- ◯ Yes
- ◯ No

Is the water transport container broken?

- ◯ Yes
- ◯ No

» Condition of water storage container

In which condition is the water storage container?

Is the water storage container clean?

- ◯ Yes
- ◯ No

Does the water storage container have a lid?

- ◯ Yes
- ◯ No

Is the water storage container broken?

- ◯ Yes
- ◯ No

What kind of toilet does the HH have on the compound?

- ◯ No latrine
- ◯ Pit latrine
- ◯ Ventilated improved latrine
- ◯ Water-sealed latrine

» Condition of the toilet

In which condition is the toilet?

Take a picture of the toilet.

Click here to upload file. (< 5MB)

Is the toilet clean?

There are no traces of faeces and dirt in the toilet

- ◯ Yes
- ◯ No

Are these material available?

- ☐ Sandals/slippers
- ☐ Drum with water
- ☐ Brush
- ☐ None of these

What kind of handwashing facilities does the HH have?

- ◯ None
- ◯ A drum with a tap
- ◯ They pour out water from a bucket

» Condition of handwashing facilities

In which conditions are the hand washing facilities?

Are the handwashing facilities in good condition?
Handwashing station is operational and not broken

- ◯ Yes
- ◯ No

Are the handwashing facilities clean?
Handwashing station has shows no signs of dirt

- ◯ Yes
- ◯ No

Is soap available?

- ◯ Yes
- ◯ No

Is water available?

- ◯ Yes
- ◯ No

» Garbage

Garbage

Can you see trash spread outside the house?

- ◯ Yes
- ◯ No

Does the household have a garbage pit to dispose garbage?

- ◯ Yes
- ◯ No

Can you see trash spread inside the house?

- ◯ Yes
- ◯ No

» Kitchen

Kitchen

Are clean dishes kept high?

- ◯ Yes
- ◯ No

Is the entirety of food covered ?

- ◯ Yes
- ◯ No

Is there dry rack to dry your utensils and dishes after washing?

- ◯ Yes
- ◯ No

Is there a significant number of flies in the kitchen (more than 10)?

if more than 10, type yes

- ◯ Yes
- ◯ No

» Personal hygiene

Personal hygiene

Is the Mother/Father wearing shoes?

Look at respondant feet

- ◯ Yes
- ◯ No

Are the Mother/Father's hands clean?

Look at respondant hand

- ◯ Yes
- ◯ No

Are the hands of child clean?

No traces of dirt visible

- ◯ Yes
- ◯ No

Can you see any dirty cloth piles lying?

No traces of dirt visible

- ◯ Yes
- ◯ No

****Please record any additional information here or in your notebook**

____

****Take GPS point of household location**

Go outside of the house, turn on the GPS button on the tablet and do not forget to switch it off afterward!

latitude (x.y °)

____

longitude (x.y °)

____

altitude (m)

____

accuracy (m)

____

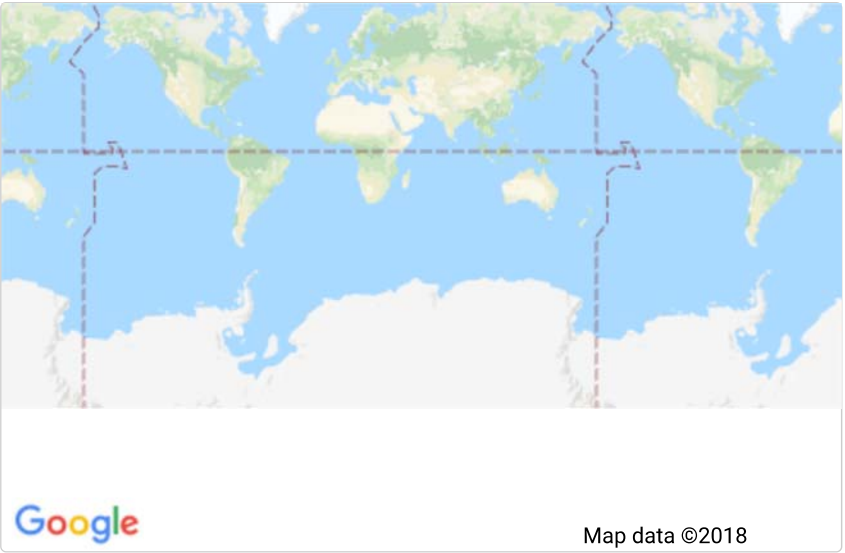

Has the water that was collected been treated in anyway?

- ○ Yes, at system level only
- ○ Yes, at household level only
- ○ Yes, at both system and household level
- ○ No treatment

Specify the treatment at the system level

- ☐ Chlorine
- ☐ Filter
- ☐ Coagulation/settlement
- ☐ UV
- ☐ Source protection / buffer zone
- ☐ Other (specify)
- ☐ Don't know/ no answer

Specify other system level treatment

____

Specify the treatment at the household level

- [ ] Chlorine
- [ ] Screening/filtration with Cloth
- [ ] Ceramic filter
- [ ] Silver coated filter
- [ ] Coagulation/settlement
- [ ] UV/SODIS
- [ ] Boiling
- [ ] Other (specify)
- [ ] Don't know / no answer

Specify other household treatment

Thank you for answering these questions!
This is the end of the interview.
Ask how far it is
