# Supplementary material for: Association of nutrition, water, sanitation and hygiene practices with children’s nutritional status, intestinal parasitic infections and diarrhoea in rural Nepal: a cross-sectional study
Source: BMC Public Health. 2020 Aug 15;20:1241. doi: 10.1186/s12889-020-09302-3 (PMC7429949; doi:10.1186/s12889-020-09302-3)
Supplement: Supplementary file 2 — Additional file 2: Table A. characteristics of the study population. [file 12889_2020_9302_MOESM2_ESM.docx]

| **Supplementary Table A**  Characteristics of the study population [N=1427] | | | | | |  |
| --- | --- | --- | --- | --- | --- | --- |
| Demographic and socioeconomic characteristics | [N (%)] | Surkhet A [n (%)] | Surkhet B [n (%)] | Dailekh [n (%)] | Accham [n (%)] | *P-* value* |
|  |  | N=356 | N=358 | N=333 | N=380 |  |
| Caregivers sociodemographic characteristics (N=1427) |  |  |  |  |  |  |
| Sex of the caregivers |  |  |  |  |  |  |
| Female | 1347 (94.4) | 337 (96.8) | 358 (98.1) | 332 (93.3) | 320 (89.4) | 0.01 |
| Male | 80 (5.6) | 11 (3.2) | 7 (1.9) | 24 (6.7) | 38 (10.6) |  |
| Age of the caregivers^a^ |  |  |  |  |  |  |
| 15-25 years | 402 (28.2) | 105 (30.2) | 120 (32.9) | 105 (29.5) | 72 (20.1) | 0.01 |
| 25-40 years | 827 (57.9) | 193 (55.5) | 215 (58.9) | 207 (58.1) | 212 (59.2) |  |
| > 40 years | 198 (13.9) | 50 (14.4) | 30 (8.2) | 44 (12.4) | 74 (20.7) |  |
| Age of the children included in the study |  |  |  |  |  |  |
| < 5 years | 1414 (99.1) | 346 (99.4) | 362 (99.2) | 353 (99.2) | 353 (98.6) | 0.70 |
| > 5 years | 13 (0.9) | 2 (0.6) | 3 (0.8) | 3 (0.84) | 5 (1.40) |  |
| Caregivers’ literacy |  |  |  |  |  |  |
| Can neither read or write | 235 (16.5) | 32 (9.2) | 12 (3.3) | 41 (11.5) | 150 (41.9) | 0.01 |
| Can read only | 24 (1.7) | 3 (0.9) | 6 (1.6) | 14 (3.9) | 1 (0.3) |  |
| Can both read or write | 1168 (81.8) | 313 (89.9) | 347 (95.1) | 301 (84.5) | 207 (57.8) |  |
| Highest education level the caregivers have completed |  |  |  |  |  |  |
| Informal education | 412 (28.9) | 49 (14.1) | 37 (10.1) | 109 (30.6) | 217 (60.6) | 0.01 |
| Primary | 484 (33.9) | 135 (38.8) | 195 ( 54.4) | 101 (28.4) | 53 (14.8) |  |
| Secondary | 362 (25.4) | 126 (36.2) | 106 (29.0) | 89 (25.0) | 41 (11.4) |  |
| College and higher | 100 (7.0) | 18 (5.2) | 25 (6.8) | 44 (12.4) | 13 (3.6) |  |
| None | 69 (4.8) | 20 (5.7) | 2 (0.5) | 13 (3.6) | 34 (9.5) |  |
| Occupation of the head of the household^b^ |  |  |  |  |  |  |
| Agriculture | 865 (60.6) | 159 (45.7) | 188 (51.5) | 250 (70.2) | 268 (74.9) | 0.01 |
| Business | 145 (10.2) | 20 (5.7) | 34 (9.3) | 57 (16.0) | 34 (9.5) | 0.01 |
| Daily labourer | 517 (36.2) | 118 (31.9) | 127 ( 34.8) | 100 (28.1) | 172 (48.0) | 0.01 |
| Private employed | 175 (12.3) | 43 (12.4) | 69 (18.9) | 47 (13.2) | 16 (4.5) | 0.01 |
| Government service | 56 (3.9) | 22 (6.3) | 17 (4.7) | 11 (3.1) | 6 (1.7) | 0.01 |
| Other independent work | 7 (0.5) | 2 (0.6) | 3 (0.8) | 1 (0.3) | 1 (0.3) | 0.68 |
| Retired with pension | 5 (0.3) | 2 (0.6) | 1 (0.3) | 2 (0.6) | 0 (0.0) | 0.55 |
| None | 12 (0.8) | 0 (0.0) | 4 (1.1) | 3 (0.8) | 5 (1.4) | 0.21 |
| Ethnicity |  |  |  |  |  |  |
| Dalit | 451 (31.6) | 81 (23.3) | 161 (44.1) | 111 (31.2) | 98 (27.4) | 0.01 |
| Janajati | 266 (18.6) | 161 (46.3) | 103 (28.2) | 1 (0.3) | 1 (0.3) |  |
| Brahmin, Chhetri, Thakuri | 704 (49.3) | 106 (30.5) | 101 (28.7) | 238 (66.8) | 259 (72.3) |  |
| Other | 6 (0.4) | 0 (0.0) | 0 (0.0) | 6 (1.7) | 0 (0.0) |  |
| Caregivers' socioeconomic status^c^ |  |  |  |  |  |  |
| Poorest | 475 (33.3) | 30 (8.6) | 31 (8.5) | 144 (40.5) | 270 (75.4) | 0.01 |
| Average | 474 (33.2) | 134 (38.5) | 131 (35.9) | 123 (34.5) | 86 (24.0) |  |
| Above average | 478 (33.5) | 184 (52.9) | 203 (55.6) | 89 (25.0) | 2 (0.6) |  |
| Floor material of the household |  |  |  |  |  |  |
| Earth | 1200 (84.1) | 255 (73.3) | 272 (74.5) | 321 (90.2) | 352 (98.3) | 0.01 |
| Cement | 227 (15.9) | 93 (26.7) | 85 (25.5) | 35 (9.8) | 6 (1.7) |  |
| Animal in the household |  |  |  |  |  |  |
| Yes | 851 (59.7) | 162 (46.5) | 174 (47.8) | 190 (53.4) | 325 (90.8) | 0.01 |
| No | 575 (40.3) | 186 (53.4) | 190 (52.2) | 166 (46.6) | 33 (9.2) |  |
| Children’s demographic characteristics |  |  |  |  |  |  |
| Sex |  |  |  |  |  |  |
| Girls | 790 (55.4) | 198 (56.9) | 196 (53.7) | 210 (59.0) | 186 (52.0) | 0.23 |
| Boys | 637 (44.6) | 150 (43.1) | 169 (46.3) | 146 (41.0) | 172 (48.0) |  |
| Age of children^d^ |  |  |  |  |  |  |
| 6 months to 5 years | 908 (63.6) | 209 (60.1) | 221 (60.5) | 232 (65.2) | 246 (68.7) | 0.05 |
| More than 5 years | 519 (36.4) | 139 (39.9) | 144 (39.4) | 124 (34.8) | 112 (31.3) |  |
| *^a^ Mean age of caregivers=30.2 (*±*8.9) years; minimum age=15 years and maximum age 70 years* | | | | | | |
| *^b^Multiple responses possible for the variable characterising occupation of the household head.* | | | |  |  |  |
| *^c^ Socio-economic status was derived from a factor analysis using principal component analysis of variables indicating the wealth index such as monthly expenditure, electricity connection at house, household ownership, rooms in the household, land ownership, and possession of household assets an electricity, radio, television, solar panel, mobile phone, bicycle, motorbike, car, fridge, watch. The score of the first factor was then divided into three categories using the k-means procedure.* | | | | | | |
| *^d^ Mean age of the children in months=50.6 (30.1) months; minimum age in months=6 months and maximum 120 months* | | | | | | |
| **P-values were obtained by χ2 test* | | | | | | |
