# Supplementary material for: Association of nutrition, water, sanitation and hygiene practices with children’s nutritional status, intestinal parasitic infections and diarrhoea in rural Nepal: a cross-sectional study
Source: BMC Public Health. 2020 Aug 15;20:1241. doi: 10.1186/s12889-020-09302-3 (PMC7429949; doi:10.1186/s12889-020-09302-3)
Supplement: Supplementary file 3 — Additional file 3: Table B. Detailed information on Children’s nutritional status in the four study sites. [file 12889_2020_9302_MOESM3_ESM.docx]

| **Supplementary Table B**  Detailed information on children’s nutritional status in the four study sites [N=1427] | | | | | | | | | | | | |
| --- | --- | --- | --- | --- | --- | --- | --- | --- | --- | --- | --- | --- |
| Nutrition and dietary variables | [n (%)] | Sex | | *P*-value* | Age | | *P*-value* | Study site | | | | *P*-value* |
|  |  | Male (n=790) | Female (n=637) |  | <6 months (n=908) | > 6 months (n=519) |  | Surkhet A [n(%)] | Surkhet B [n (%)] | Dailekh [n (%)] | Accham [n(%)] |  |
| Child ever breastfed | 1422 (99.6) | 787 (99.6) | 635 (99.7) | 0.83 | 906 (99.8) | 516 (99.4) | 0.27 | 345 (99.1) | 364 (99.7) | 356 (100.0) | 357 (99.7) | 0.26 |
| Total months of breastfeeding of child |  |  |  |  |  |  |  |  |  |  |  |  |
| <6 months | 19 (1.3) | 9 (1.1) | 10 (1.6) | 0.30 | 14 (1.5) | 5 (1.0) | **0.01** | 9 (2.6) | 6 (1.6) | 4 (1.2) | 0 (0.0) | **0.01** |
| 6-12 months | 773 (54.2) | 416 (52.7) | 357 (56.0) |  | 552 (60.8) | 221 (42.6) |  | 175 (50.3) | 181 (49.6) | 208 (58.4) | 209 (58.4) |  |
| >12 months | 635 (44.5) | 365 (46.2) | 270 (42.4) |  | 342 (37.7) | 293 (56.4) |  | 164 (47.1) | 178 (48.8) | 144 (40.5) | 149 (41.6) |  |
| Complementary feeding for the child started <6months |  |  |  |  |  |  |  |  |  |  |  |  |
| Yes | 1384 (97.0) | 766 (97.0) | 618 (97.0) | 0.95 | 883 (97.3) | 501 (96.5) | 0.45 | 318 (91.4) | 357 (97.8) | 355 (99.7) | 354 (98.9) | **0.01** |
| No | 43 (3.0) | 24 (3.0) | 19 (3.0) |  | 25 (2.7) | 18 (3.5) |  | 30 (8.6) | 8 (2.2) | 1 (0.3) | 4 (1.1) |  |
| Child's meal consumption per day |  |  |  |  |  |  |  |  |  |  |  |  |
| < 2 meals | 40 (2.8) | 24 (3.0) | 16 (2.5) | 0.55 | 33 (3.6) | 7 (1.4) | **0.01** | 22 (3.2) | 10 (2.7) | 4 (1.1) | 15 (4.2) | 0.09 |
| >3 meals | 1387 (97.2) | 766 (97.0) | 621 (97.5) |  | 875 (96.4) | 512 (98.6) |  | 337 (96.8) | 355 (97.3) | 352 (98.9) | 343 (95.8) |  |
| Child receiving food supplements in addition to regular meal |  |  |  |  |  |  |  |  |  |  |  |  |
| Yes | 618 (43.3) | 351 (44.5) | 267 (41.9) | 0.33 | 403 (44.4) | 215 (41.5) | 0.29 | 166 (47.8) | 179 (49.0) | 159 (44.7) | 114 (31.8) | **0.01** |
| No | 808 (56.7) | 438 (55.5) | 370 (58.1) |  | 505 (55.6) | 303 (58.5) |  | 181 (52.2) | 186 (51.0) | 197 (55.3) | 244 (68.2) |  |
| Dietary Diversity Scores (DDS)^a^ based on food consumed in past one week |  |  |  |  |  |  |  |  |  |  |  |  |
| 1 | 158 (11.1) | 95 (12.0) | 63 (9.9) | 0.23 | 102 (11.2) | 56 (10.8) | 0.58 | 63 (18.1) | 16 (4.4) | 65 (18.2) | 14 (3.9) | **0.01** |
| 2 | 147 (10.3) | 85 (10.8) | 62 (9.7) |  | 88 (9.7) | 59 (11.4) |  | 78 (22.4) | 29 (7.9) | 28 (7.9) | 12 (3.3) |  |
| 3 | 170 (11.9) | 83 (10.5) | 87 (13.7) |  | 109 (12.0) | 61 (11.8) |  | 59 (16.9) | 52 (14.2) | 33 (9.3) | 26 (7.3) |  |
| 4 | 154 (10.8) | 79 (10.0) | 75 (11.8) |  | 92 (10.1) | 62 (12.0) |  | 48 (13.8) | 35 (9.6) | 31 (8.7) | 40 (11.2) |  |
| 5 | 160 (11.2) | 93 (11.8) | 67 (10.5) |  | 106 (11.7) | 54 (10.4) |  | 26 (7.5) | 37 (10.1) | 36 (10.1) | 61 (17.0) |  |
| 6 | 162 (11.3) | 80 (10.1) | 82 (12.9) |  | 101 (11.1) | 61 (11.8) |  | 25 (7.2) | 62 (17.0) | 39 (11.0) | 36 (10.1) |  |
| 7 | 158 (11.1) | 87 (11.0) | 71 (11.2) |  | 105 (11.6) | 53 (10.2) |  | 26 (7.5) | 47 (12.9) | 30 (8.4) | 55 (15.4) |  |
| 8 | 158 (11.1) | 94 (11.9) | 64 (10.0) |  | 94 (10.3) | 64 (12.3) |  | 11 (3.2) | 44 (12.1) | 42 (11.8) | 61 (17.0) |  |
| 9 | 160 (11.2) | 94 (11.9) | 66 (10.4) |  | 111 (12.2) | 49 (9.4) |  | 12 (3.4) | 43 (11.8) | 52 (14.6) | 53 (14.8) |  |
| *^a^ the dietary diversity scores are based on the type of food consumed in the week preceding the survey. The food was categorized into nine food groups such as (i) starchy staples (ii) beans/peas/lentils, (iii) nuts (iv) dairy products (v) meat/fish (vi) eggs (vii) leafy green vegetables; (viii) other vegetables and (ix) fruits. The details of the food consumption can be found in the Table D, supplementary materials.* | | | | | | | | | | | | |
