# Supplementary material for: Association of nutrition, water, sanitation and hygiene practices with children’s nutritional status, intestinal parasitic infections and diarrhoea in rural Nepal: a cross-sectional study
Source: BMC Public Health. 2020 Aug 15;20:1241. doi: 10.1186/s12889-020-09302-3 (PMC7429949; doi:10.1186/s12889-020-09302-3)
Supplement: Supplementary file 4 — Additional file 4: Table C. Nutrition provided to children between 6 months and 10 years. [file 12889_2020_9302_MOESM4_ESM.doc]

| **Supplementary Table C**  **Nutrition provided to children between 6 months and 10 years** | | | | | | |
| --- | --- | --- | --- | --- | --- | --- |
| Dietary variables | [n (%)] | Surkhet A [n (%)] | Surkhet B [n (%)] | Dailekh [n (%)] | Accham [n (%)] | *P*-value* |
| **Type of food supplements provided (N=1427)** |  |  |  |  |  |  |
| Cereals^a^ | 594 (41.6) | 153 (44.0) | 169 (46.3) | 158 (44.4) | 114 (31.8) | 0.01 |
| Fruit^b^ | 5 (0.4) | 4 (1.2) | 0 (0.0) | 1 (0.3) | 0 (0.0) | 0.03 |
| Meat^c^ | 2 (0.1) | 1 (0.3) | 1 (0.3) | 0 (0.0) | 0 (0.0) | 0.57 |
| Eggs | 9 (0.6) | 1 (1.4) | 3 (0.8) | 0 (0.0) | 1 (0.3) | 0.08 |
| Legumes and seed^d^ | 59 (4.1) | 24 (6.9) | 23 (6.3) | 5 (1.4) | 7 (2.0) | 0.01 |
| Milk and milk products^e^ | 44 (3.1) | 17 (4.9) | 15 (4.1) | 3 (0.8) | 9 (2.5) | 0.01 |
| Oil and fat^f^ | 2 (0.1) | 0 (0.0) | 1 (0.3) | 0 (0.0) | 1 (0.3) | 0.58 |
| Sweets^g^ | 3 (0.2) | 3 (0.9) | 0 (0.0) | 0 (0.0) | 0 (0.0) | 0.03 |
| Spices, condiments and beverages^h^ |  |  |  |  |  |  |
| Type of regular food (week prior to the survey) (N=1427) |  |  |  |  |  |  |
| **Starchy staples^i^** |  |  |  |  |  |  |
| Three times per day | 215 (15.1) | 64 (18.4) | 80 (21.9) | 0 (0.0) | 71 (19.8) | 0.01 |
| Twice per day | 821 (57.5) | 198 (56.9) | 197 (54.0) | 260 (73.0) | 166 (46.4) |  |
| Once per day | 389 (27.3) | 85 (24.4) | 88 (24.1) | 95 (26.7) | 121 (33.8) |  |
| Two times per week | 1 (0.1) | 0 (0.0) | 0 (0.0) | 1 (0.3) | 0 (0.0) |  |
| Sometimes | 1 (0.1) | 1 (0.3) | 0 (0.0) | 0 (0.0) | 0 (0.0) |  |
| **Beans, peas or lentils** |  |  |  |  |  |  |
| Three times per day | 42 (2.9) | 15 (4.3) | 14 (3.8) | 4 (1.1) | 9 (2.5) | 0.01 |
| Twice per day | 583 (40.9) | 128 (36.8) | 136 (37.3) | 195 (54.8) | 124 (34.6) |  |
| Once per day | 726 (50.9) | 178 (51.2) | 201 (55.1) | 147 (41.3) | 200 (55.9) |  |
| Every second day | 28 (2.0) | 12 (3.5) | 4 (1.1) | 1 (0.3) | 11 (3.1) |  |
| Two times per week | 12 (0.8) | 4 (1.2) | 1 (0.3) | 1 (0.3) | 6 (1.7) |  |
| Once per week | 3 (0.2) | 1 (0.3) | 2 (0.6) | 0 (0.0) | 0 (0.0) |  |
| Less than once per week | 3 (0.2) | 0 (0.0) | 1 (0.3) | 1 (0.3) | 1 (0.3) |  |
| Sometimes | 29 (2.0) | 9 (2.6) | 6 (1.6) | 7 (2.0) | 7 (2.0) |  |
| Not at all | 1 (0.1) | 1 (0.3) | 0 (0.0) | 0 (0.0) | 0 (0.0) |  |
| **Nuts** |  |  |  |  |  |  |
| Twice per day | 3 (0.2) | 0 (0.0) | 0 (0.0) | 2 (0.6) | 1 (0.3) | 0.01 |
| Once per day | 11 (0.8) | 6 (1.7) | 5 (1.4) | 0 (0.0) | 0 (0.0) |  |
| Every second day | 1 (0.3) | 0 (0.0) | 0 (0.0) | 0 (0.0) | 0 (0.0) |  |
| Two times per week | 1 (0.1) | 1 (0.3) | 0 (0.0) | 0 (0.0) | 0 (0.0) |  |
| Once per week | 12 (0.8) | 8 (2.3) | 2 (0.6) | 1 (0.3) | 1 (0.3) |  |
| Less than once per week | 3 (0.2) | 1 (0.3) | 2 (0.6) | 0 (0.0) | 0 (0.0) |  |
| Sometimes | 1129 (79.1) | 268 (77.0) | 325 (89.0) | 301 (84.6) | 235 (65.6) |  |
| Not at all | 267 (18.7) | 63 (18.1) | 31 (8.5) | 52 (14.6) | 121 (33.8) |  |
| **Milk and milk products** |  |  |  |  |  |  |
| Twice per day | 140 (9.8) | 13 (3.7) | 37 (10.1) | 33 (9.3) | 57 (15.9) | 0.01 |
| Once per day | 277 (19.4) | 75 (21.5) | 75 (20.6) | 65 (18.3) | 62 (17.3) |  |
| Every second day | 27 (1.9) | 8 (2.3) | 11 (3.0) | 4 (1.1) | 4 (1.1) |  |
| Two times per week | 48 (3.4) | 11 (3.2) | 10 (2.7) | 17 (4.8) | 10 (2.8) |  |
| Once per week | 132 (9.2) | 20 (5.8) | 44 (12.1) | 42 (11.8) | 26 (7.3) |  |
| Less than once per week | 65 (4.6) | 4 (1.2) | 12 (3.3) | 13 (3.6) | 36 (10.1) |  |
| Sometimes | 708 (49.6) | 204 (58.6) | 173 (47.4) | 175 (49.2) | 156 (43.6) |  |
| Not at all | 30 (2.1) | 13 (3.7) | 3 (0.8) | 7 (2.0) | 7 (2.0) |  |
| **Meat or fish** |  |  |  |  |  |  |
| Twice per day | 8 (0.6) | 1 (0.3) | 2 (0.5) | 5 (1.4) | 0 (0.0) |  |
| Once per day | 50 (3.5) | 6 (1.7) | 19 (5.2) | 22 (6.2) | 3 (0.8) |  |
| Every second day | 120 (8.4) | 24 (6.9) | 38 (10.4) | 32 (9.0) | 26 (7.3) |  |
| Two times per week | 230 (16.1) | 104 (29.9) | 66 (18.1) | 41 (11.5) | 19 (5.3) |  |
| Once per week | 606 (42.5) | 143 (41.1) | 161 (44.1) | 169 (47.5) | 133 (37.1) |  |
| Less than once per week | 131 (9.2) | 23 (6.6) | 18 (4.9) | 33 (9.3) | 57 (15.9) |  |
| Sometimes | 275 (19.3) | 45 (12.9) | 61 (16.7) | 53 (14.9) | 116 (32.4) |  |
| Not at all | 7 (0.5) | 2 (0.6) | 0 (0.0) | 1 (0.3) | 4 (1.1) |  |
| **Eggs** |  |  |  |  |  |  |
| Three times per day | 1 (0.1) | 0 (0.0) | 1 (0.3) | 0 (0.0) | 0 (0.0) | 0.01 |
| Twice per day | 4 (0.3) | 1 (0.3) | 1 (0.3) | 2 (0.6) | 0 (0.0) |  |
| Once per day | 58 (4.1) | 13 (3.7) | 30 (8.2) | 14 (3.9) | 1 (0.3) |  |
| Every second day | 46 (3.2) | 9 (2.6) | 27 (7.4) | 9 (2.5) | 1 (0.3) |  |
| Two times per week | 68 (4.8) | 27 (7.8) | 23 (6.3) | 14 (3.9) | 4 (1.1) |  |
| Once per week | 75 (5.3) | 25 (7.2) | 20 (5.5) | 20 (5.6) | 10 (2.8) |  |
| Less than once per week | 11 (0.8) | 4 (1.1) | 2 (0.5) | 2 (0.3) | 3 (0.8) |  |
| Sometimes | 1054 (73.9) | 245 (70.4) | 260 (71.2) | 260 (73.0) | 289 (80.7) |  |
| Not at all | 110 (7.7) | 24 (6.9) | 1 (0.3) | 35 (9.8) | 50 (14.0) |  |
| **Leafy green vegetables** |  |  |  |  |  |  |
| Twice per day | 266 (18.6) | 22 (6.3) | 52 (14.2) | 88 (24.7) | 104 (29.0) | 0.01 |
| Once per day | 511 (35.8) | 86 (24.7) | 173 (47.4) | 98 (27.5) | 154 (43.0) |  |
| Every second day | 129 (9.0) | 32 (9.2) | 37 (10.1) | 38 (10.7) | 22 (6.1) |  |
| Two times per week | 109 (7.6) | 60 (17.2) | 19 (5.2) | 9 (2.5) | 21 (5.9) |  |
| Once per week | 69 (4.8) | 21 (6.0) | 10 (2.7) | 14 (3.9) | 24 (6.7) |  |
| Less than once per week | 25 (1.7) | 17 (4.9) | 3 (0.8) | 3 (0.8) | 2 (0.6) |  |
| Sometimes | 316 (22.1) | 109 (31.3) | 71 (19.4) | 106 (29.8) | 30 (8.4) |  |
| Not at all | 2 (0.1) | 1 (0.3) | 0 (0.0) | 0 (0.0) | 1 (0.3) |  |
| **Other vegetables** |  |  |  |  |  |  |
| Three times per day | 8 (0.6) | 1 (0.3) | 0 (0.0) | 3 (0.8) | 4 (1.1) |  |
| Twice per day | 314 (22.0) | 41 (11.8) | 113 (31.0) | 87 (24.4) | 73 (20.4) |  |
| Once per day | 738 (51.7) | 140 (40.2) | 180 (49.3) | 173 (48.6) | 245 (68.4) |  |
| Every second day | 56 (3.9) | 21 (6.0) | 22 (6.0) | 4 (1.1) | 9 (2.5) |  |
| Two times per week | 32 (2.2) | 13 (3.7) | 8 (2.2) | 5 (1.4) | 6 (1.7) |  |
| Once per week | 19 (1.3) | 7 (2.0) | 7 (1.9) | 4 (1.1) | 1 (0.3) |  |
| Less than once per week | 12 (0.8) | 9 (2.6) | 2 (0.3) | 2 (0.6) | 0 (0.0) |  |
| Sometimes | 244 (17.1) | 115 (33.0) | 33 (9.0) | 78 (21.9) | 18 (5.0) |  |
| Not at all | 4 (0.3) | 1 (0.3) | 1 (0.3) | 0 (0.0) | 2 (0.6) |  |
| **Fruit** |  |  |  |  |  |  |
| Twice per day | 6 (0.4) | 1 (0.3) | 0 (0.0) | 4 (1.1) | 1 (0.3) | 0.01 |
| Once per day | 18 (1.3) | 5 (1.4) | 4 (1.1) | 8 (2.2) | 1 (0.3) |  |
| Every second day | 8 (0.6) | 4 (1.1) | 2 (0.5) | 2 (0.6) | 0 (0.0) |  |
| Two times per week | 21 (1.5) | 13 (3.7) | 2 (0.5) | 6 (1.7) | 0 (0.0) |  |
| Once per week | 25 (1.7) | 12 (3.4) | 2 (0.5) | 9 (2.5) | 2 (0.6) |  |
| Less than once per week | 15 (1.0) | 6 (1.7) | 6 (1.6) | 0 (0.0) | 3 (0.8) |  |
| Sometimes | 1295 (90.7) | 302 (86.8) | 349 (95.6) | 314 (88.2) | 330 (92.2) |  |
| Not at all | 39 (2.7) | 5 (1.4) | 0 (0.0) | 13 (3.6) | 21 (5.9) |  |
| *^a^ Cereals: corn/maize, rice, wheat, millet or any other grains (e.g. bread, noodles, porridge or other grain products) + local food products* | | | | | | |
| *^b^ Fruit: any type of fruit (fresh or dried)* |  |  |  |  |  |  |
| *^c^ Meat: beef, pork, lamb, goat, rabbit, game, chicken, duck, other birds,* | | | |  |  |  |
| *^d^ Legumes and seed: dried beans, dried peas, lentils, nuts, seed or food made from these (e.g. peanut butter)* | | | | |  |  |
| *^e^ Milk and milk products: milk, cheese, yoghurt or other milk products* | | |  |  |  |  |
| *^f^ Oil and fat: oil, fat or butter added to food or used for cooking* | | |  |  |  |  |
| *^g^ Sweets: sugar, honey, sweetened soda or sweetened juice drinks, sugary foods such as chocolate, candies, cookies and cakes* | | | | | | |
| *^h^ Spices, condiments, beverages: spices (black pepper, salt), condiments (soy sauce, hot sauce), coffee, tea, alcoholic beverages* | | | | | | |
| *^i^ Starchy staples/cereals* |  |  |  |  |  |  |
