# Supplementary material for: Association of nutrition, water, sanitation and hygiene practices with children’s nutritional status, intestinal parasitic infections and diarrhoea in rural Nepal: a cross-sectional study
Source: BMC Public Health. 2020 Aug 15;20:1241. doi: 10.1186/s12889-020-09302-3 (PMC7429949; doi:10.1186/s12889-020-09302-3)
Supplement: Supplementary file 5 — Additional file 5: Table D. Water supply, water handling and water quality. [file 12889_2020_9302_MOESM5_ESM.docx]

| **Supplementary Table D**  Water supply, water handling and water quality [N=1427] | | | | | | | |
| --- | --- | --- | --- | --- | --- | --- | --- |
| Variables | [N (%)] | | Surkhet A [n (%)] | Surkhet B [n (%)] | Dailekh [n (%)] | Accham [n (%)] | *P*-value* |
| Involvement in the water supply system in the community | 1352 (94.7) | | 284 (81.6) | 357 (97.8) | 354 (99.4) | 357 (99.7) | 0.01 |
| Main drinking water source |  | |  |  |  |  |  |
| Piped water in the house or yard | 296 (20.7) | | 15 (4.3) | 268 (73.4) | 9 (2.5) | 4 (1.1) | 0.01 |
| Piped water in the village | 1077 (75.5) | | 292 (83.9) | 97 (26.5) | 340 (95.5) | 348 (97.2) |  |
| Open source^a^ | 25 (1.7) | | 20 (5.7) | 0 (0.0) | 5 (1.4) | 0 (0.0) |  |
| Protected source^b^ | 20 (1.4) | | 14 (4.0) | 0 (0.0) | 0 (0.0) | 6 (1.7) |  |
| Unmanaged piped system | 4 (0.3) | | 2 (0.6) | 0 (0.0) | 2 (0.6) | 0 (0.0) |  |
| River, stream or canal | 5 (0.3) | | 5 (1.4) | 0 (0.0) | 0 (0.0) | 0 (0.0) |  |
| Other water sources used for drinking water^c^ |  | |  |  |  |  |  |
| Piped water in the house or yard | 113 (7.9) | | 3 (0.9) | 106 (29.0) | 4 (1.1) | 0 (0.0) | 0.01 |
| Piped water in the village | 320 (22.4) | | 105 (30.2) | 23 (6.3) | 65 (18.3) | 127 (35.3) | 0.01 |
| Open source | 32 (2.2) | | 30 (8.6) | 2 (0.5) | 0 (0.0) | 0 (0.0) | 0.01 |
| Protected source | 7 (0.5) | | 5 (1.4) | 0 (0.0) | 0 (0.0) | 2 (0.6) | 0.02 |
| Unmanaged piped system | 1 (0.1) | | 1 (0.3) | 0 (0.0) | 0 (0.0) | 0 (0.0) | 0.38 |
| River/Stream | 14 (1.0) | | 14 (4.02) | 0 (0.0) | 0 (0.0) | 0 (0.0) | 0.01 |
| Bottled water | 1 (0.1) | | 1 (0.3) | 0 (0.0) | 0 (0.0) | 0 (0.0) | 0.38 |
| Time required for fetching drinking water |  | |  |  |  |  |  |
| <5 minutes | 57 (4.0) | | 18 (5.2) | 34 (9.3) | 1 (0.3) | 4 (1.1) | 0.01 |
| 5-15 minutes | 882 (61.8) | | 185 (53.2) | 308 (84.4) | 165 (46.3) | 224 (62.6) |  |
| 15 minutes to 60 minutes | 453 (31.7) | | 141 (40.5) | 23 (6.3) | 169 (47.5) | 120 (33.5) |  |
| > 60 minutes | 35 (2.4) | | 4 (1.1) | 0 (0.0) | 21 (5.9) | 10 (2.8) |  |
| Functioning of main drinking water supply |  | |  |  |  |  |  |
| Functioning well | 1,300 (91.1) | | 283 (81.3) | 353 (96.7) | 338 (94.9) | 326 (91.1) | 0.01 |
| Functioning but regularly | 126 (8.8) | | 64 (18.4) | 12 (3.3) | 18 (5.1) | 32 (8.9) |  |
| Not functioning | 1 (0.1) | | 1 (0.3) | 0 (0.0) | 0 (0.0) | 0 (0.0) |  |
| Main water supply not functioning in the last 6 months |  | |  |  |  |  |  |
| Yes | 47 (3.3) | | 25 (7.2) | 21 (5.7) | 0 (0.0) | 1 (0.3) | 0.01 |
| No | 1380 (96.7) | | 323 (92.8) | 344 (94.2) | 356 (100) | 357 (99.7) |  |
| Caregivers’ opinion on factors that makes drinking water unsafe^d^ |  | |  |  |  |  |  |
| Open unprotected source | 1155 (80.9) | | 297 (85.3) | 312 (85.5) | 332 (93.3) | 214 (59.8) | 0.01 |
| Unmanaged system | 714 (50.0) | | 128 (36.8) | 169 (46.3) | 235 (66.0) | 182 (50.8) |  |
| Open defecation | 835 (58.5) | | 231 (66.4) | 229 (62.7) | 266 (74.7) | 109 (30.4) |  |
| Settlement above source | 324 (22.7) | | 56 (16.1) | 80 (21.9) | 112 (31.5) | 76 (21.2) |  |
| Deforestation | 50 (3.5) | | 17 (4.9) | 21 (5.7) | 10 (2.8) | 2 (0.6) |  |
| Don’t know | 121 (8.5) | | 21 (6.0) | 33 (9.0) | 10 (2.8) | 57 (15.9) |  |
| Knowledge about water treatment methods^d^ |  | |  |  |  |  |  |
| Boiling | 816 (57.2) | | 203 (58.3) | 244 (66.8) | 220 (61.8) | 149 (41.6) | 0.01 |
| Filtration with a cloth | 448 (31.4) | | 91 (26.1) | 151 (41.4) | 124 (34.8) | 82 (22.9) | 0.01 |
| Flocculation and sedimentation | 24 (1.7) | | 5 (1.4) | 10 (2.7) | 8 (2.2) | 1 (0.3) | 0.06 |
| Chlorination | 116 (8.1) | | 29 (8.3) | 39 (10.7) | 32 (9.0) | 16 (4.5) | 0.02 |
| Sodis | 94 (6.6) | | 35 (10.1) | 33 (9.0) | 16 (4.5) | 10 (2.8) | 0.01 |
| Water filter | 842 (59.0) | | 239 (68.7) | 260 (71.2) | 209 (58.7) | 134 (37.4) | 0.01 |
| Other | 1 (0.1) | | 0 (0.0) | 0 (0.0) | 1 (0.3) | 0 (0.0) | 0.39 |
| Do not know any way | 460 (32.2) | | 82 (23.6) | 73 (20.0) | 111 (31.2) | 194 (54.2) | 0.01 |
| Water treatment methods used^d^ |  | |  |  |  |  |  |
| Boiling | 58 (4.1) | | 22 (6.3) | 31 (8.5) | 3 (0.8) | 2 (0.6) | 0.01 |
| Filtration with a cloth | 34 (2.4) | | 10 (2.9) | 24 (6.4) | 0 (0.0) | 0 (0.0) | 0.01 |
| Flocculation and sedimentation | 3 (0.2) | | 3 (0.9) | 0 (0.0) | 0 (0.0) | 0 (0.0) | 0.03 |
| Chlorination | 1 (0.1) | | 1 (0.3) | 0 (0.0) | 0 (0.0) | 0 (0.0) | 0.38 |
| Sodis | 2 (0.1) | | 1 (0.3) | 1 (0.3) | 0 (0.0) | 0 (0.0) | 0.57 |
| Use of filter | 137 (9.6) | | 50 (14.4) | 31 (8.5) | 52 (14.6) | 4 (1.1) | 0.01 |
| Cleaning the container for drinking water transport |  | |  |  |  |  |  |
| Yes | 1416 (99.2) | | 347 (99.7) | 363 (99.4) | 355 (99.7) | 351 (98.0) | 0.03 |
| No | 11 (0.8) | | 1 (0.3) | 2 (0.6) | 1 (0.3) | 351 (98.0) |  |
| Cleaning the container for drinking water transport |  | |  |  |  |  |  |
| Every day | 1206 (85.2) | | 280 (80.7) | 334 (92.0) | 309 (87.0) | 283 (80.6) | 0.01 |
| Every second day | 177 (12.5) | | 53 (15.3) | 21 (5.8) | 40 (11.3) | 63 (17.9) |  |
| At least once per week | 27 (1.9) | | 13 (3.7) | 6 (1.6) | 4 (1.1) | 4 (1.1) |  |
| Less than once per week | 6 (0.4) | | 1 (0.3) | 2 (0.5) | 2 (0.6) | 1 (0.3) |  |
| Materials used to clean water transport container |  | |  |  |  |  |  |
| Water/water and sand | 390 (27.5) | | 120 (34.6) | 104 (28.6) | 37 (10.4) | 129 (36.7) | 0.01 |
| Always with soap or ash | 429 (30.3) | | 84 (24.2) | 141 (38.8) | 108 (30.4) | 96 (27.3) |  |
| Sometime with soap or ash | 597 (42.2) | | 143 (41.2) | 118 (32.5) | 210 (59.1) | 126 (35.9) |  |
| Cleaning the container for drinking water storage (n=79) |  | |  |  |  |  |  |
| Yes | 78 (98.7) | | 67 (98.5) | 7 (100.0) | 4 (100.0) | 0 (0.0) | 0.92 |
| No | 1 (1.3) | | 1 (1.3) | 0 (0.0) | 0 (0.0) | 0 (0.0) |  |
| Frequency of cleaning water storage container |  | |  |  |  |  |  |
| Every day | 53 (67.9) | | 44 (65.7) | 6 (85.7) | 3 (75.0) | 0 (0.0) | 0.01 |
| Every second day | 18 (23.1) | | 18 (26.9) | 0 (0.0) | 0 (0.0) | 0 (0.0) |  |
| At least once per week | 6 (7.7) | | 5 (7.5) | 1 (14.3) | 0 (0.0) | 0 (0.0) |  |
| Less than once per week | 1 (1.3) | | 0 (0.0) | 0 (0.0) | 1 (25.0) | 0 (0.0) |  |
| Cleaning the container for drinking water storage |  | |  |  |  |  |  |
| Water/water and sand | 24 (30.8) | | 23 (34.3) | 1 (14.3) | 0 (0.0) | 0 (0.0) | 0.01 |
| Always with soap or ash | 17 (21.8) | | 10 (14.9) | 6 (85.7) | 1 (25.0) | 0 (0.0) |  |
| Sometime with soap or ash | 37 (47.4) | | 34 (50.7) | 0 (0.0) | 3 (75.0) | 0 (0.0) |  |
| **Drinking water quality at the point of collection** |  | |  |  |  |  |  |
| Total coliform bacteria (n=1136) |  | |  |  |  |  |  |
| 0 CFU^e^/100 mL | 7 (0.6) | | 0 (0.0) | 0 (0.0) | 7 (2.0) | 0 (0.0) | 0.01 |
| 1-10 CFU/100 mL | 18 (1.6) | | 1 (0.5) | 0 (0.0) | 17 (4.9) | 0 (0.0) |  |
| 10-100 CFU/100 mL | 20 (1.8) | | 4 (2.1) | 5 (1.6) | 9 (2.6) | 2 (0.7) |  |
| 100-1000 CFU/100 mL | 884 (77.8) | | 101 (53.4) | 274 (88.7) | 258 (74.1) | 251 (86.6) |  |
| >1000 CFU/100 mL | 207 (18.2) | | 83 (43.9) | 30 (9.7) | 57 (16.4) | 37 (12.8) |  |
| *Escherichia coli* |  | |  |  |  |  |  |
| 0 CFU/100 mL | 74 (6.5) | | 26 (13.7) | 2 (0.6) | 45 (13.0) | 1 (0.3) | 0.01 |
| 1-10 CFU/100 mL | 315 (27.8) | | 94 (49.5) | 83 (27.0) | 74 (21.3) | 64 (22.1) |  |
| 10-100 CFU/100 mL | 574 (50.6) | | 59 (31.1) | 201 (65.3) | 139 (40.1) | 175 (60.3) |  |
| 100-1000 CFU/100 mL | 153 (13.5) | | 6 (3.2) | 20 (6.5) | 84 (24.2) | 43 (14.8) |  |
| >1000 CFU/100 mL | 19 (1.7) | | 5 (2.6) | 2 (0.7) | 5 (1.4) | 7 (2.4) |  |
| **Drinking water quality at the point of use** |  | |  |  |  |  |  |
| Total coliform bacteria (n=1257) |  | |  |  |  |  |  |
| 0 CFU/100 mL | 16 (1.3) | | 12 (4.0) | 2 (0.9) | 1 (0.3) | 0 (0.0) | 0.01 |
| 1-10 CFU/100 mL | 27 (2.2) | | 15 (5.1) | 8 (2.5) | 4 (1.2) | 0 (0.0) |  |
| 10-100 CFU/100 mL | 40 (3.2) | | 15 (5.1) | 8 (2.5) | 15 (4.3) | 2 (0.7) |  |
| 100-1000 CFU/100 mL | 743 (59.1) | | 100 (33.7) | 223 (68.8) | 218 (62.5) | 202 (70.4) |  |
| >1000 CFU/100 mL | 431 (34.3) | | 155 (52.2) | 82 (25.3) | 111 (31.8) | 83 (28.9) |  |
| *Escherichia coli* (n=1260) |  | |  |  |  |  |  |
| 0 CFU/100 mL | 60 (4.8) | | 33 (11.0) | 13 (4.0) | 13 (3.7) | 1 (0.4) | 0.01 |
| 1-10 CFU/100 mL | 206 (16.4) | | 77 (25.7) | 41 (12.7) | 57 (16.3) | 31 (10.8) |  |
| 10-100 CFU/100 mL | 643 (51.0) | | 142 (47.3) | 187 (57.7) | 150 (43.0) | 164 (57.1) |  |
| 100-1000 CFU/100 mL | 292 (23.2) | | 32 (10.7) | 71 (21.9) | 115 (33.0) | 74 (25.8) |  |
| >1000 CFU/100 mL | 59 (4.7) | | 16 (5.3) | 12 (3.7) | 14 (4.0) | 17 (5.9) |  |
| Hygiene condition of water transport container^f^ (observation) |  | |  |  |  |  |  |
| Lower category | 871 (61.0) | | 247 (71.0) | 256 (70.1) | 221 (62.1) | 147 (41.1) | 0.01 |
| Middle category | 214 (15.0) | | 33 (9.5) | 34 (9.3) | 42 (11.8) | 105 (29.3) |  |
| Higher category | 342 (24.0) | | 68 (19.5) | 75 (20.6) | 93 (26.1) | 106 (29.6) |  |
| Hygiene condition of water storage container^g^ (observation) |  | |  |  |  |  |  |
| Lower category | 864 (60.5) | | 250 (71.8) | 255 (69.9) | 215 (60.4) | 144 (40.2) | 0.01 |
| Middle category | 212 (14.9) | | 28 (8.1) | 35 (9.6) | 44 (12.4) | 105 (29.3) |  |
| Higher category | 351 (24.6) | | 70 (20.1) | 75 (20.5) | 97 (27.2) | 109 (30.4) |  |
| *^a^ Open source refers to unprotected dug wells, ponds and unprotected springs.* | | | | | | | |
| *^b^* *Protected source were refers to protected wells and protected springs.* | |  |  |  |  |  |  |
| *^c^* *Multiple responses were possible for other water sources of drinking water.* | | | |  |  |  |  |
| ^d^ *Multiple responses were possible for the variables characterising the caregivers opinion on factors that makes drinking water unsafe, knowledge about water treatment methods and the treatment methods being used and containers used for the transport of drinking water* | | | | | | | |
| ^e^ *CFU=Colony forming unit* | |  |  |  |  |  |  |
| ^f^*A new variable for the hygiene condition of the container used for the transport of drinking water was created using factor analysis with three conceptually similar categorical variables of : (i) water transport container is clean; (ii) water transport container has a lid; and (iii) water transport container is broken. The hygiene condition of the drinking water transport container was then categorised into three categories of lower, middle and better hygiene.* | | | | | | | |
| ^g^ *A new variable for the hygiene condition of the container used for the storage of drinking water was created using factor analysis with three conceptually similar categorical variables of: (i) water storage container is clean; (ii) water storage container has a lid; and (iii) water storage container is broken. The hygiene condition of the drinking water storage container was then categorised into three categories of lower, middle and better hygiene.* | | | | | | | |
| **P-values were obtained by χ2 test* | |  |  |  |  |  |  |
