# Supplementary material for: Association of nutrition, water, sanitation and hygiene practices with children’s nutritional status, intestinal parasitic infections and diarrhoea in rural Nepal: a cross-sectional study
Source: BMC Public Health. 2020 Aug 15;20:1241. doi: 10.1186/s12889-020-09302-3 (PMC7429949; doi:10.1186/s12889-020-09302-3)
Supplement: Supplementary file 6 — Additional file 6: Table E. Child health, health seeking behaviour and awareness on health protecting behaviours. [file 12889_2020_9302_MOESM6_ESM.docx]

| **Supplementary Table E**  Child health, health seeking behaviour and awareness on health protecting behaviours [N=1427] | | | | | | |
| --- | --- | --- | --- | --- | --- | --- |
| Health variables | [N (%)] | Surkhet A [n (%)] | Surkhet B [n (%)] | Dailekh [n (%)] | Accham [n (%)] | *P*-value* |
| **Child health^a^** |  |  |  |  |  |  |
| Surveyed child sick within past 7 days | 712 (49.9) | 156 (44.8) | 192 (52.6) | 178 (50.0) | 186 (52.0) | 0.15 |
| Child suffered from illness in past 7 days^b^ |  |  |  |  |  |  |
| Fever | 565 (39.6) | 119 (34.2) | 152 (41.6) | 137 (38.5) | 157 (43.8) | 0.05 |
| Cough | 555 (38.9) | 123 (35.3) | 168 (46.0) | 126 (35.4) | 138 (38.5) | 0.01 |
| Respiratory illness | 217 (15.2) | 53 (15.2) | 62 (17.0) | 39 (11.0) | 63 (17.6) | 0.06 |
| Diarrhoea^c^ | 235 (16.5) | 36 (10.3) | 59 (16.2) | 69 (19.4) | 71 (19.8) | 0.01 |
| Blood in stool | 46 (3.2) | 3 (0.9) | 18 (4.9) | 5 (1.4) | 20 (5.6) | 0.01 |
| Mucus in stool | 53 (3.7) | 4 (1.1) | 22 (6.0) | 8 (2.2) | 19 (5.3) | 0.01 |
| Blood in urine | 9 (0.6) | 0 (0.0) | 6 (1.6) | 0 (0.0) | 3 (0.8) | 0.01 |
| **Health seeking behaviour** |  |  |  |  |  |  |
| Seeking medical advice for illness of surveyed child |  |  |  |  |  |  |
| Yes | 599 (84.0) | 129 (82.7) | 169 (87.6) | 147 (82.6) | 154 (82.8) | 0.48 |
| No | 114 (16.0) | 27 (17.3) | 24 (12.4) | 31 (17.4) | 32 (17.2) | 0.48 |
| Seek medical advice/treatment from^d^ |  |  |  |  |  |  |
| Hospital | 76 (5.3) | 14 (4.0) | 15 (4.1) | 28 (7.9) | 19 (5.3) | 0.08 |
| Health centre/health post | 285 (20.0) | 48 (13.8) | 77 (21.1) | 69 (19.4) | 91 (25.4) | 0.01 |
| Community health worker | 27 (1.9) | 0 (0.0) | 0 (0.0) | 6 (1.7) | 21 (5.9) | 0.01 |
| Pharmacy | 256 (17.9) | 67 (19.2) | 85 (23.3) | 66 (18.5) | 38 (10.6) | 0.01 |
| Self-treatment and traditional medicine | 3 (0.2) | 1 (0.3) | 1 (0.3) | 1 (0.3) | 0 (0.0) | 0.80 |
| Other | 5 (0.3) | 5 (1.4) | 0 (0.0) | 0 (0.0) | 0 (0.0) | 0.01 |
| Reason for not seeking medical advice^e^ |  |  |  |  |  |  |
| "I do not have good access to a health facility" | 2 (1.8) | 2 (7.4) | 0 (0.0) | 0 (0.0) | 0 (0.0) | 0.01 |
| "I do not have money to go to the health facility" | 24 (21.2) | 1 (3.7) | 1 (4.3) | 7 (22.6) | 15 (46.9) | 0.01 |
| "I prefer self-treatment or traditional medicine" | 8 (7.1) | 0 (0.0) | 5 (21.7) | 0 (0.0) | 3 (9.4) | 0.01 |
| " It was not necessary to go to the health facility" | 70 (61.9) | 17 (63.0) | 17 (73.9) | 22 (71.0) | 14 (43.7) | 0.01 |
| Other | 9 (8.0) | 7 (25.9) | 0 (0.0) | 2 (6.4) | 0 (0.0) | 0.01 |
| **KAP survey^f^** |  |  |  |  |  |  |
| Causes of diarrhoeal diseases |  |  |  |  |  |  |
| Some pathogens | 71 (5.0) | 22 (6.3) | 13 (3.6) | 34 (9.5) | 2 (0.6) | 0.01 |
| Faecal pathogens | 706 (49.5) | 173 (49.7) | 173 (47.4) | 198 (55.6) | 162 (45.3) | 0.04 |
| Dirty hands | 706 (49.5) | 173 (49.7) | 173 (47.4) | 198 (55.6) | 162 (45.2) | 0.04 |
| Dirty food | 1145 (80.2) | 315 (90.5) | 296 (81.1) | 307 (86.2) | 227 (63.4) | 0.01 |
| Dirty water | 1180 (82.7) | 298 (85.6) | 295 (80.8) | 319 (89.6) | 268 (74.9) | 0.01 |
| Explanation does not correspond with real cause | 90 (6.3) | 5 (1.4) | 23 (6.3) | 11 (3.1) | 51 (14.2) | 0.01 |
| Chances of getting sick drinking untreated water |  |  |  |  |  |  |
| Very low | 3 (0.2) | 2 (0.6) | 1 (0.3) | 0 (0.0) | 0 (0.0) | 0.01 |
| Rather low | 44 (3.1) | 8 (2.3) | 12 (3.3) | 0 (0.0) | 24 (6.7) |  |
| Average | 94 (6.6) | 29 (8.3) | 33 (9.0) | 4 (1.1) | 28 (7.8) |  |
| Rather high | 300 (21.0) | 107 (30.8) | 49 (13.4) | 47 (13.2) | 97 (27.1) |  |
| Very high | 986 (69.1) | 202 (58.0) | 270 (74.0) | 305 (85.7) | 209 (58.4) |  |
| Heard of "intestinal parasite" |  |  |  |  |  |  |
| Yes | 301 (21.1) | 109 (31.3) | 66 (18.1) | 84 (23.6) | 42 (11.7) | 0.01 |
| No | 1126 (78.9) | 239 (68.7) | 299 (81.9) | 272 (76.4) | 316 (88.3) |  |
| Knowledge on protective measures against intestinal parasites^g^ |  |  |  |  |  |  |
| Wash hands with soap | 158 (11.1) | 70 (20.1) | 23 (6.3) | 53 (14.9) | 12 (3.4) | 0.01 |
| Cut finger nails | 76 (5.3) | 21 (6.0) | 14 (3.8) | 33 (9.3) | 8 (2.2) | 0.01 |
| Wear pants, trousers | 49 (3.4) | 26 (7.5) | 6 (1.6) | 12 (3.4) | 5 (1.5) | 0.01 |
| Wash fruits and vegetables before consumption | 26 (1.8) | 1 (0.3) | 9 (2.5) | 13 (3.7) | 3 (0.8) | 0.01 |
| Wear shoes | 71 (5.0) | 23 (6.6) | 13 (3.6) | 27 (7.6) | 8 (2.2) | 0.01 |
| Drink clean water | 237 (16.6) | 86 (24.7) | 54 (14.8) | 77 (21.6) | 20 (5.6) | 0.01 |
| Regular deworming | 159 (11.1) | 59 (17.0) | 37 (10.1) | 35 (9.8) | 28 (7.8) | 0.01 |
| ^a^ *Health of the youngest surveyed child* |  |  |  |  |  |  |
| ^b^ *Multiple answers possible* |  |  |  |  |  |  |
| ^c^ *Passage of liquid stool more than 3 times per day* |  |  |  |  |  |  |
| ^d^ *Multiple responses possible for seeking medical advice or treatment* | | |  |  |  |  |
| ^e^ *Multiple responses possible for reasons for not seeking medical advice* | | |  |  |  |  |
| ^f^ *KAP: knowledge attitude and practices survey* |  |  |  |  |  |  |
| ^g^ *Multiple responses possible for knowledge on protective measures against intestinal parasites* | | |  |  |  |  |
